# Detection of *Solanum betaceum Cav* fruit maturity using YOLO11-based deep learning

**DOI:** 10.3389/fpls.2026.1880899

**Published:** 2026-09-10

**Authors:** Lenin Quiñones Huatangari, Héctor Vladimir Vásquez Pérez, Lamberto Valqui-Valqui, Jose Manuel Palomino Ojeda, David Saravia, William Bardales Escalante, Gelver Silva, Antony Chavez-Jalk, Omer Cruz Caro, Leandro Valqui

**Affiliations:** 1 Escuela Profesional de Ingeniería de Ciencia de Datos e Inteligencia Artificial, Universidad Nacional Toribio Rodríguez de Mendoza de Amazonas, Chachapoyas, Peru; 2 Laboratorio de Agrostología, Instituto de Investigación en Ganadería y Biotecnología, Universidad Nacional Toribio Rodríguez de Mendoza de Amazonas, Chachapoyas, Peru; 3 Laboratorio de Agrostología, Instituto de Investigación en Ganadería y Biotecnología, Universidad Nacional Toribio Rodríguez de Mendoza de Amazonas, Chachapoyas, Peru; 4 Data Science Research Institute, Universidad Nacional de Jaén, Jaén, Peru; 5 Estación Experimental Agraria Amazonas, Instituto Nacional de Innovacion Agraria, La Molina, Peru; 6 Oficina de Gestion de la Calidad, Universidad Nacional Toribio Rodríguez de Mendoza de Amazonas, Chachapoyas, Peru

**Keywords:** computer vision, deep learning, fruit ripeness evaluation, object detection, tree tomato

## Abstract

The tree tomato (*Solanum betaceum Cav*.) is a Solanaceae fruit native to South America with nutritional, functional, and economic value. However, assessing fruit maturity and making harvest-related decisions continue to rely primarily on manual visual inspection, which can be subjective and inconsistent under field conditions. The objective of this study was to evaluate the performance of YOLO11-based object detection models for the automatic detection of the ripeness of *Solanum betaceum* fruits in a real-world agricultural setting. To this end, a dataset of 200 images captured with smartphones was used, in which 1,361 fruits were annotated with bounding boxes corresponding to green and ripe fruits. The YOLO11n, YOLO11s, and YOLO11m models were trained with an input resolution of 640. For each setting, cross-validation (k = 5) was used, and data augmentation was appliedss-validation (k=5) for each setting and applying data augmentation to the training subsets. YOLO11m achieved the best overall performance, as it combined high values fr precision, recall, and mAP. On the test set, YOLO11m achieved an precision of 0.9878 ± 0.0055, a recall of 0.9750 ± 0.148, and an mAP50 of 0.9618 ± 0.0188 for green fruit, as well as a precision of 0.9950 ± 0.0041, a recall of 0.9958 ± 0.0039, and a mAP50 of 0.9920 ± 0.0047 for ripe fruits. YOLO11s also demonstrated competitive performance, particularly in the detection of ripe fruits, with an mAP50 of 0.9894 ± 0.0070 and an mAP50–95 of 0.9754 ± 0.0081, positioning it as an efficient alternative given its substantially lower computational cost. In contrast, YOLO11n, the lightest architecture, showed the lowest performance for green fruits (mAP50-95: 0.8557 ± 0.0611), reflecting the limitations of its 7representational capacity.

## 1 Introduction

The tamarillo, or tree tomato (*S. betaceum* Cav.), is a solanaceous plant native to South America (Caeiro et al., 2023), a small tree cultivated in various regions of the world (Rito et al., 2023) that is susceptible to nematode attack (Coyago-Cruz et al., 2023) and one of the most widely consumed fruits in tropical countries (Ndisanze and Koca, 2023). The fruit has antioxidant properties and contains vitamins and nutrients that are vital for human health (Astiti Asih et al., 2023), may help alleviate cognitive decline and memory loss (Imania et al., 2024), is recognized for its complete nutritional profile and valued for its ethnobotanical uses (Huang and Huang, 2024), and has economic potential due to its nutritional value (Murillo-Gómez et al., 2017).

The nutritional and functional value of *S. betaceum* reinforces the importance of improving harvest and maturity assessment practices. Previous studies have reported that its fruits and by-products contain bioactive compounds such as polyphenols, carotenoids, anthocyanins, dietary fiber, vitamins, and minerals, which contribute to its antioxidant potential and economic value (MaChado et al., 2024; Rohilla and Mahanta, 2024). However, fruit maturity is a dynamic process associated with visible color changes and internal quality attributes, including soluble solids, acidity, firmness, pigmentation, and colorimetric parameters (Diep et al., 2020; Hu et al., 2023). In practice, maturity assessment still depends largely on manual visual inspection, which can be subjective and inconsistent under field conditions (Garillos-Manliguez and Chiang, 2021). Therefore, image-based detection offers a practical alternative for identifying maturity stages more objectively and supporting timely harvest decisions (Anjali et al., 2024).

Computer vision and deep learning (DL) constitute useful tools for automating maturity assessment (Ching et al., 2018; Kim, 2016; Zhou et al., 2025), as they allow digital fruit images to be transformed into numerical matrices capable of representing visual information associated with color, texture, shape, and surface variation (Garillos-Manliguez and Chiang, 2021). Unlike traditional methods based on manual feature extraction, DL models can learn visual patterns directly from images, which improves the identification of maturity stages in scenarios involving gradual pigmentation changes, variable illumination, fruit overlap, or complex natural backgrounds (Cong et al., 2023; Ma et al., 2024; Yang et al., 2024). In particular, detectors based on convolutional neural networks and YOLO-type architectures have shown advanced performance in locating and classifying fruits according to their maturity condition (Isiaka, 2024; Mienye and Swart, 2024; Talaei Khoei et al., 2023; Yu et al., 2019), due to their ability to process images and recognize visual differences between classes (Wei et al., 2024; Xiao et al., 2023).

Deep learning (DL), as a branch of machine learning and artificial intelligence, enables models to learn hierarchical visual representations from image data and has become a relevant computational approach for real-time computer vision applications (Alzubaidi et al., 2021; Mienye and Swart, 2024; Mishra et al., 2021; Patra et al., 2023; Sarker, 2021). In the field of agriculture, the utilisation of digital light technology for the identification and assessment of fruit maturity has garnered significant attention. Manual visual grading of fruit maturity is a laborious, subjective, and inconsistent process. In contrast, the timely and accurate detection of maturity using DL technology provides crucial support for effective harvest planning and significantly reduces postharvest losses (Ajil et al., 2023; Karegowda et al., 2024; Mi and Yan, 2024; Zhang, 2023). In the realm of object detection methodologies, YOLO-based models have demonstrated a notable aptitude for field applications, a consequence of their capacity for real-time, single-stage detection. Previous reviews have underscored the increasing adoption of these models in the context of fruit detection and recognition tasks (Xiao et al., 2023). Nonetheless, the detection of *S. betaceum* maturity remains a visually challenging process. This is due to the gradual nature of the colour transitions, the variability in fruit size and shape, the partial occlusion of the fruit by leaves or branches, the natural changes in illumination, the presence of shadows, and the complex backgrounds that are present under real production conditions.

Nevertheless, fruit detection in real plantation environments represents a significant undertaking within the domain of agricultural computer vision, due to the inherent technical challenges it poses (Gong and Wu, 2025). In order to enhance the capacity for automatic detection of ripening fruit in conditions of uneven lighting and occlusion, a range of computer vision methodologies has been employed to detect and locate the fruit in question. As demonstrated in the study Chen et al. (2021), convolutional neural networks (CNNs) have demonstrated considerable efficacy in a range of agricultural contexts. In the specific case of fruit such as dates, CNNs have been employed to facilitate the identification and classification of the fruit according to its ripeness (Pérez-Pérez et al., 2021) and to classify tomato fruit according to shape, group, color, and defects (Faria et al., 2025). The ripeness of blueberries was detected, counted, and evaluated using YOLOv8 and YOLOv9 (Deng et al., 2024); the ripeness of citrus fruits was identified based on the R-LBP algorithm and the YOLO-CIT model (Wang et al., 2024); and the Tiny YOLO-v4 model was improved to identify the ripening period of pineapples (Cuong et al., 2022).

However, although computer vision and deep learning have advanced in fruit detection and maturity assessment, their application to *Solanum betaceum* Cav. remains limited, particularly under real field conditions. Therefore, this study aimed to evaluate the performance of YOLO11s, YOLO11n, and YOLO11m models for detecting and visually assessing tree tomato maturity, using 200 smartphone images and 1,217 fruits classified as green and ripe. The contribution of this study lies in showing that lightweight YOLO11 models can become an accessible and objective alternative to support harvest decision-making, technical assistance, and the modernization of Andean-tropical agricultural systems.

## 2 Materials and methods

**Figure 1:**
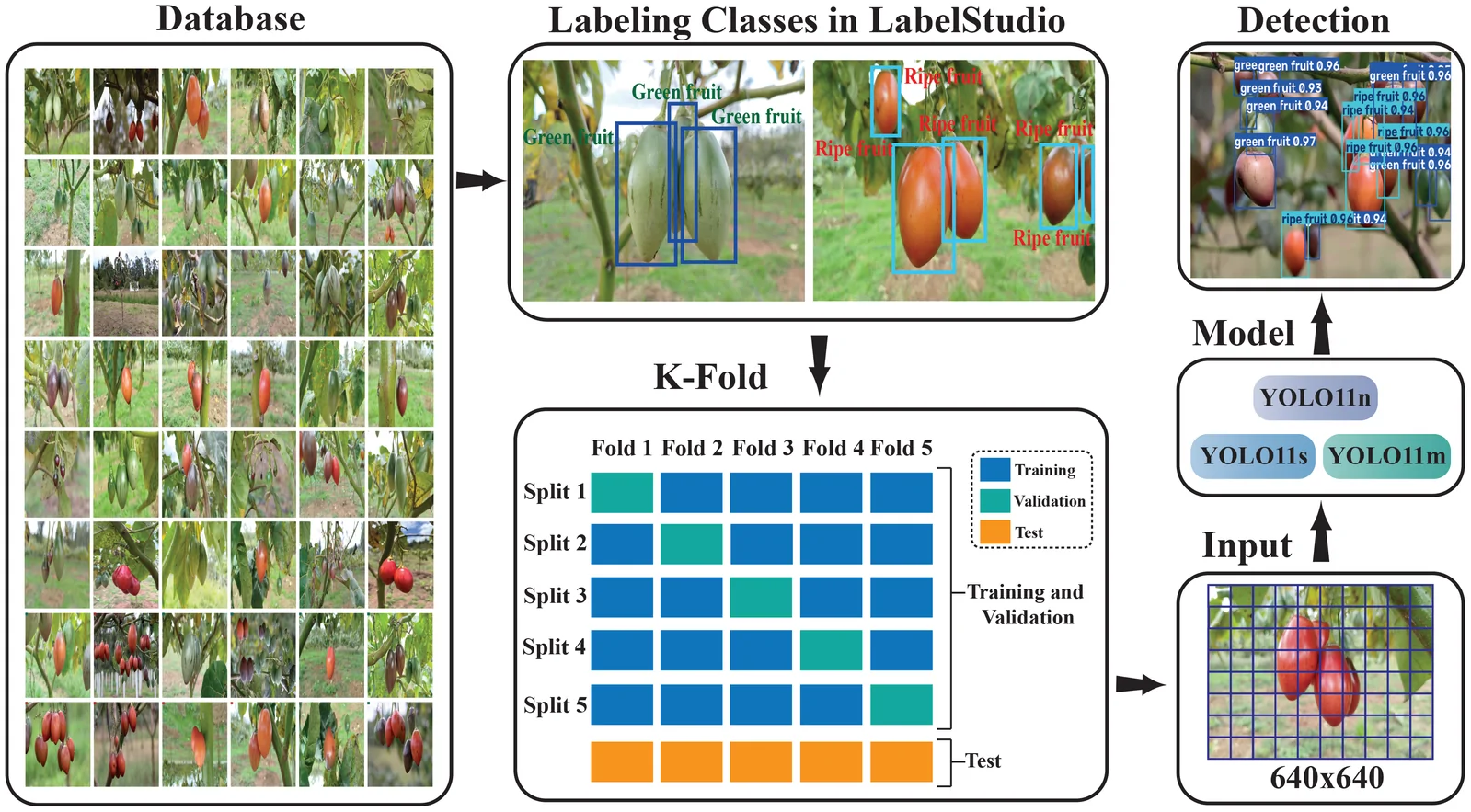
Workflow for the YOLO11-based detection and visual maturity assessment of *S. betaceum Cav*.

The proposed methodology consisted of several stages (See **Figure 1**): data acquisition and annotation, experimental design, class balancing, model development, model training, and performance evaluation. Initially, a dataset consisting of 200 field-collected images of *Solanum betaceum* Cav. was manually annotated, dividing them into two maturity classes (green fruit and ripe fruit). Subsequently, the original dataset was divided into independent training and test subsets, after which a five-fold cross-validation (K = 5) was performed. To address the class imbalance, a hybrid balancing strategy was applied that combined offline oversampling through data augmentation with undersampling—applied only to the training sub- sets—thereby preventing data leakage. Next, three pre-trained variants of YOLO11 (YOLO11n, YOLO11s, and YOLO11m) were trained under identical experimental conditions, using optimized hyperparameters and in-line data augmentation to improve the model’s robustness and generalization. Finally, the trained models were evaluated on the test set using standard object detection metrics, including precision, recall, mAP50, and mAP50–95, which allowed for a comprehensive comparison of their performance in detecting the fruits of *S. betaceum Cav*.

### 2.1 Dataset

**Figure 2:**
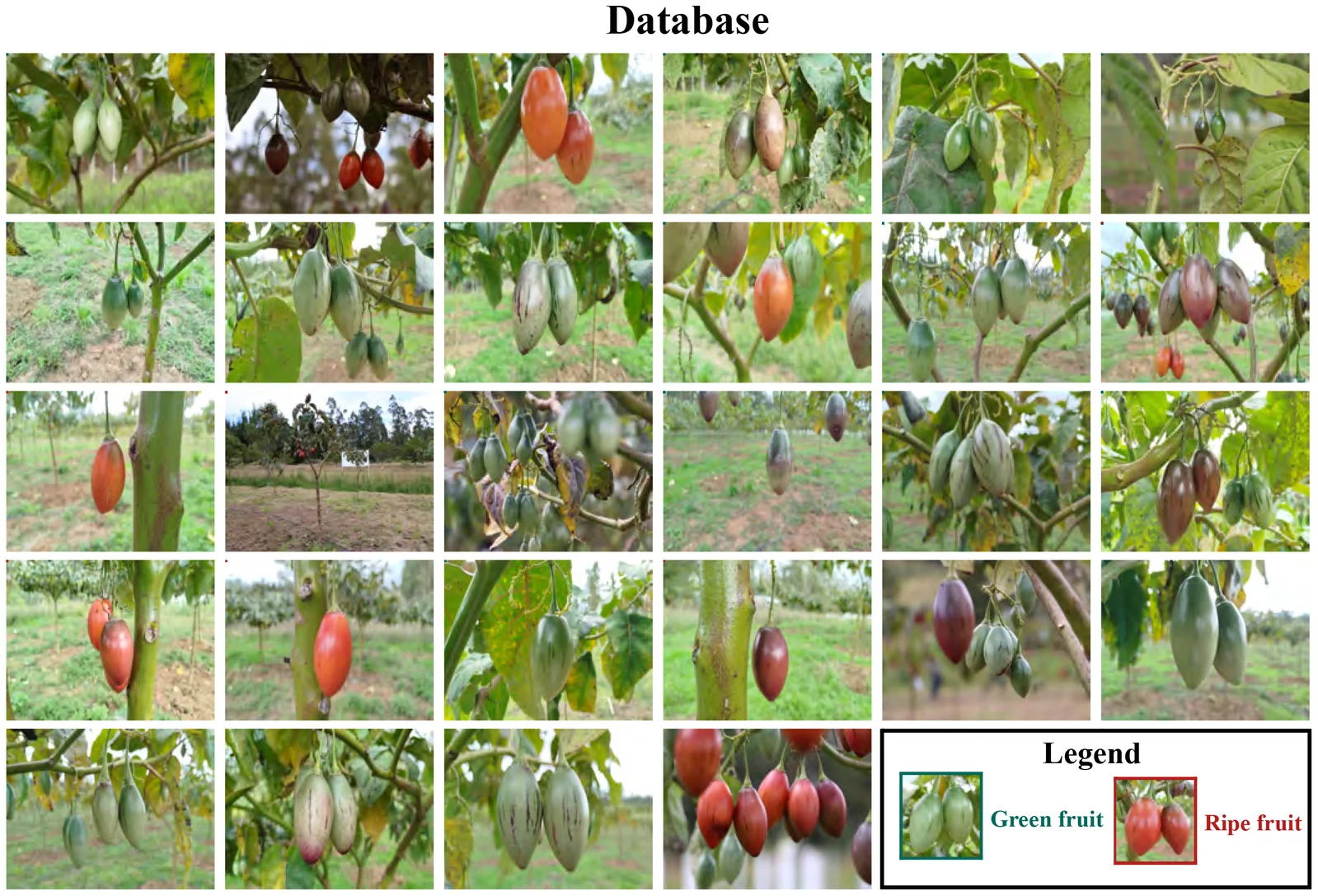
Sample images of *S. betaceum Cav* plants captured under field conditions.

The original dataset consisted of 200 color images of *S. betaceum* Cav. captured using smartphone cameras at the Amazonas agricultural experimental station of the National Institute of Agricultural Innovation (INIA), located in Fundo San Juan-Chachapoyas, from February to May 2025. The images were obtained in advance of the peak harvest season and included fruits at varying stages of ripeness. Data acquisition took place under natural field conditions, accounting for differing light levels, viewing angles, and shooting distances. Consequently, the dataset comprised images ranging from individual branches to entire shrub canopies. This increased the variability necessary for robust fruit detection under real-world agricultural conditions (See **Figure 2**).

All images were subjected to manual annotation by a team of qualified professionals, who adhered to a standardised protocol for annotation. This process was conducted using the Label Studio software, version 1.23.0 (HumanSignal, 2025) Fruit ripeness was classified into two categories—unripe and ripe fruits based on their external color. Although the colour of fruit naturally varies from green to reddish-orange during the process of ripening, this binary classification system was found to facilitate the annotation process and reduce the occurrence of labelling inconsistencies. Given that a significant proportion of fruits occupied relatively small regions of the image, it was necessary to perform annotations at a magnification of at least 300%. Subsequently, each annotation underwent a separate quality-control procedure to identify and correct potential labeling errors before being included in the final dataset. The verified annotations were exported from Label Studio in YOLO format and converted into text files compatible with the YOLO training framework.

**Figure 3:**
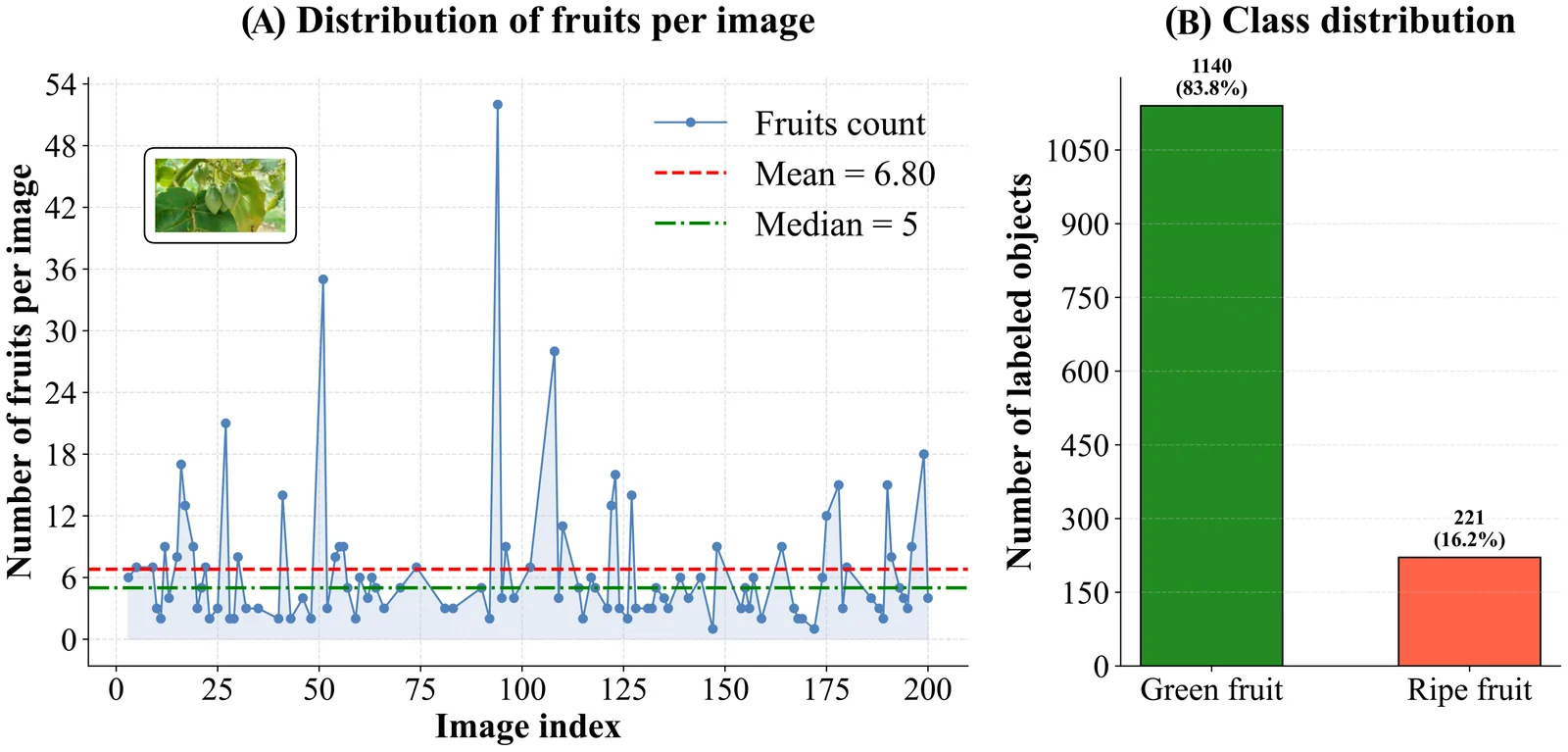
Distribution of *S. betaceum Cav.* fruits from the original database. **(A)** Distribution of the number of fruits per image, including the mean and median. **(B)** Distribution of labeled objects by class: green fruitA and ripe fruit.

The dataset contained 1,361 annotated fruit examples, including 1,140 green fruits (83.8%) and 221 ripe fruits (16.2%). It had an average of 6.80 fruits per image (median = 5), with an approximate average of 5.7 green fruits and 1.1 ripe fruits per image. This marked imbalance between classes reflects the fact that the images were captured during the early stages of fruit ripening, when ripe fruits were, naturally, less abundant (See **Figure 3**).

### 2.2 Detection of *S. betaceum Cav*

#### 2.1.1 Experimental design

To ensure an unbiased evaluation and prevent data leakage, the original dataset, consisting of 200 annotated images, was randomly divided into five folds (k=5). In each fold, 20 images were set aside as an independent test set, while the remaining 180 images were used for model development, divided into 160 images for training and 20 images for validation. Data augmentation was applied exclusively to the images in the training subset of each fold, incorporating between 96 and 113 augmented images depending on the fold, while the validation and test images remained unaugmented.

The test subsets provided the evaluation conditions for each variant of the YOLO11 model, allowing for a comparison of their performance across independent test partitions. For each fold, the Precision, Recall, mAP50, and mAP50–95 metrics were calculated independently on their corresponding test subset. Subsequently, the results were aggregated using the mean and standard deviation of the metrics obtained across the five folds. Therefore, these statistics summarize the variability in performance across the five evaluation partitions (Sa et al., 2016; Tian et al., 2019).

#### 2.2.2 Class balancing and data augmentation

To mitigate the class imbalance in the training data, a hybrid balancing strategy was adopted that combined oversampling and undersampling (Buslaev et al., 2020; Nath et al., 2020; Shorten and Khoshgoftaar, 2019). The balancing procedure was applied exclusively after data partitioning and only to the training subset of each fold. During the oversampling phase, the minority class in each training fold was expanded through offline data augmentation, using horizontal flipping, vertical flipping, random brightness adjustment (0.6–1.4), random zoom (0.8–1.2), random cropping of the edges (0–10%), and the addition of Gaussian noise (*µ* = 0, *σ* = 8) (Ghiasi et al., 2020; Shorten and Khoshgoftaar, 2019). A fixed random seed (seed = 42) was used to ensure reproducibility. Geometric transformations automatically updated the corresponding coordinates of the bounding boxes, preserving consistency between the transformed images and their annotations. Subsequently, the majority class in the training subset was subsampled to reduce class imbalance.

#### 2.2.3 YOLO models

YOLO algorithms, developed by Joseph Redmon and colleagues, detect multiple objects simultaneously. They use a single neural network to process input images and generate output bounding boxes. Each bounding box includes a confidence score for each class, enabling rapid object detection (Chunnapiya and Visutsak, 2025); it is widely used because of its detection precision, good generalization, open-source code, and speed (Kanna et al., 2024), and has pioneered real-time object detection, from version v5 to v8, constantly improving its speed and performance metrics (Rajeev et al., 2025). Since its first version in 2015, YOLO has evolved significantly: YOLOv1 introduced the single-stage detection approach. YOLO v2 and v3 improved precision with techniques such as batch normalization, anchor boxes, and multi-scale detection (Ali and Zhang, 2024). YOLOv4 and v5 further optimized speed and precision, making them popular in industrial applications (Wang et al., 2021). YOLOv7 and YOLOv8 incorporate modern and efficient architectures, such as transformers and decoupled heads, achieving state-of-the-art precision and speed (Wang et al., 2023).

YOLO11 is the latest evolution in the family of real-time object detection models developed by Ultralytics. It represents a significant leap in precision, speed, and computational efficiency. It introduces a backbone and neck design that improves feature extraction, enabling accurate detection even in complex tasks such as segmentation and oriented detection (Sapkota et al., 2025). Despite its increased precision, YOLO11m uses 22% fewer parameters than YOLOv8m, maintaining an excellent balance between performance and speed. It supports multiple computer vision tasks: object detection, instance segmentation, image classification, and oriented object detection. It is designed to run efficiently on both edge devices and in the cloud, with support for NVIDIA GPUs. Like previous versions, YOLO11 comes in variants such as YOLO11n (nano), YOLO11s (small), YOLO11m (medium), YOLO11l (large), and YOLO11x (extra-large), tailored to different precision and speed requirements.

#### 2.2.4 Model training

Three variants of the YOLO11 architecture (YOLO11n, YOLO11s, and YOLO11m) were used, employing the corresponding pre-trained weights provided by Ultralytics. All input images were resized to 640 ×640 pixels, preserving sufficient visual information for fruit detection while maintaining computational efficiency during training. The images were converted and stored in JPEG format. All experiments were conducted on the Lightning AI cloud platform using Python 3.12.11, PyTorch 2.8+cu128, the Ultralytics library (version 8.4.99), and an NVIDIA H100 GPU with 80 GB of memory. Each model was trained independently for 100, 200, and 300 epochs, using a batch size of 32 and an input image resolution of 640 × 640 pixels.

The models were optimized using a custom hyperparameter configuration based on the literature (Jia et al., 2023; Wang et al., 2025, 2024), employing the AdamW optimizer with an initial learning rate of 0.001, a final learning rate factor of 0.01, cosine scheduling of the learning rate, a weight decay of (5 × 10*^−^*^4^), and a warm-up phase of three epochs. To improve the model’s robustness and generalization, a comprehensive data augmentation strategy was applied that explicitly modified Ultralytics’ default hyperparameters, including HSV color adjustments, geometric transformations, horizontal flipping, and Mosaic, MixUp, and Copy-Paste augmentations. In addition, computational performance was improved by enabling RAM caching, eight parallel data-loading processes, and training with automatic mixed-precision (AMP) to accelerate convergence while optimizing memory usage (See **Table 1**).

**Table 1:** Training configuration and hyperparameters used for the YOLO11 models.

Parameter group	Configuration
Models	YOLO11n.pt, YOLO11s.pt and YOLO11m.pt (pre-trained weights)
Training	Epochs = [100, 200, 300], Batch = 32, Image size = 640 × 640
Optimization	AdamW (lr = 0.001, lrf = 0.01, Weight decay = 5×10^-4^, Cosine learning rate scheduler, Warm-up = 3 epochs)
Data augmentation	HSV (0.015, 0.7, 0.4); Rotation = 10°; Translation = 0.1; Scale = 0.5 [0.5,1.5]; Shear = 2°; Horizontal flip = 0.5; Mosaic = 1.0; MixUp = 0.1; Copy-Paste = 0.1
Computational settings	Cache = RAM; Workers = 8; Automatic Mixed Precision (AMP) = Enabled

#### 2.2.5 Online data augmentation

In addition to the offline data augmentation strategy adopted for class balancing (Section 2.2.2), YOLO11 performs online data augmentation during training. Data augmentation is a regularization strategy that artificially increases the diversity of the training dataset without the need to collect new images, thereby improving the generalization capacity of the model (Shorten and Khoshgoftaar, 2019). In YOLO11n, these transformations are applied dynamically during each training iteration, so that the model does not process the same representation of an image across different epochs (Jocher et al., 2023). The configuration adopted in the present study combines three categories: chromatic perturbations, geometric transformations, and image composition techniques.

##### 2.2.5.1 Perturbations in the hue-saturation-valuecolor space

Chromatic transformations operate in the HSV color space, which separates hue, saturation, and value, thereby facilitating the simulation of variable lighting conditions. The hue is randomly shifted by ±5.4° (*hsv_h = 0.015*), introducing subtle chromatic variations without altering the visual identity of the object. Saturation and brightness are scaled within the intervals [0.3, 1.7] and [0.6, 1.4], respectively (*hsv_s = 0.7; hsv_v = 0.4*), emulating cameras of different quality and heterogeneous exposure conditions.

##### 2.2.5.2 Geometric transformations

Geometric transformations simultaneously modify the image and the bounding box annotations, preserving spatial consistency. Random rotation of ±10° (*degrees* = 10), translation of up to ±10% along both axes (translate = 0.1), and scaling within the interval [0.5, 1.5] (*scale = 0.5*) were applied. The latter parameter is particularly relevant for detecting objects at different distances, as it encourages the model to develop invariance to apparent object size. In addition, shearing of ±2° (*shear = 2*) was incorporated to simulate oblique viewpoints. Horizontal flipping was enabled with a probability of 0.5 (*fliplr = 0.5*), effectively increasing dataset diversity for objects without functional asymmetry between the left and right sides.

##### 2.2.5.3 Image composition techniques

The mosaic technique (*mosaic = 1.0*), enabled for all samples, combines four images from the training dataset into a single image through a 2 × 2 spatial concatenation (Bochkovskiy et al., 2020). This transformation systematically exposes the model to partially visible objects and multiple background contexts at the same time, increasing its sensitivity to small object instances. With a probability of 0.1, *MixUp* (*mixup = 0.1*) linearly combines two images and their labels, acting as a regularizer that smooths decision boundaries (Zhang et al., 2017). Finally, Copy Paste (*copy_paste = 0.1*) inserts segmented objects from source images into target images, increasing the frequency of underrepresented classes and diversifying occlusion configurations (Ghiasi et al., 2020). Given that the training image dataset contains *N* samples and training extends over *E epochs*, the model effectively processes *N × E* distinct variations, whose stochastic combination makes the exact repetition of any augmented sample statistically unlikely.

#### 2.2.6 Performance metrics

The detection performance of *S. betaceum Cav* was evaluated in terms of the common metrics of precision, recall (Soto-Osorio et al., 2024), and mAP@50 for the green fruit and ripe fruit classes, calculated using Equations 1–3:

(1) $$Precision\;\left(\%\right)\;=\;\frac{\#TP}{\#TP\;+\;\#FP}\;\times \;100$$

(2) $$Recall\left(\%\right)\;=\;\frac{\#TP}{\#TP\;+\;\#FN}\;\times \;100$$

(3) $$mAP50\left(\%\right)\;=\frac{1}{N}\;\sum_{i=1}^{N}{\left(AP@50\right)}_{i}\;\times \;100$$

where #TP, #FP, #TN, and #FN represent the number of true positives, false positives, true negatives, and false negatives, respectively (Anuyah et al., 2024). A true positive prediction corresponds to a detection assigned with the correct class label and satisfying the predefined confidence and Intersection over Union (IoU) thresholds. In this study, the default values were adopted, namely a confidence threshold of 0.25 and an IoU threshold of 0.5, for the calculation of precision and recall.

The mAP@50 metric was calculated as the sum of the Average Precision (AP) values obtained for each class using an IoU threshold of 0.5, divided by the total number of classes (*N)* (Bochkovskiy et al., 2020). In this study, the number of classes was two, corresponding to ripe and green fruits. Since mAP50 integrates both precision and recall, it was considered the primary evaluation metric for assessing the overall multi-class detection performance of the models.

## 3 Results

The study demonstrates the feasibility of using object detection models from the YOLO11 family for the automated detection and assessment of the ripeness of *S. betaceum* Cav. fruits under real-world field conditions, laying the groundwork for the development of automated harvest assistance systems. The YOLO11n, YOLO11s, and YOLO11m models were evaluated for the detection of ripe and unripe *Solanum betaceum* fruits under real-world field conditions.

### 3.1 Database

The images encompassed variations in lighting, distance, fruit position, vegetation in the background, branches, leaves, and potential occlusions, which allowed for an evaluation of the models’ performance in a real-world agricultural setting. The validation and test subsets contain only original images, ensuring that the evaluation metrics reflect performance on real, unseen data, and only the training subset was expanded. The **Table 2** shows the distribution of images per fold (k=5), for a split of 160 images for training, 20 for validation, and 20 for testing. After performing data augmentation, the number of augmented images added to the training subset ranged from 96 to 113, depending on the fold, resulting in total training sizes ranging from 256 to 273 images.

**Table 2:** Image distribution by fold in cross-validation (K = 5).

Fold	Train	Image augmentation	Total train	Validation	Test
1	160	113	273	20	20
2	160	96	256	20	20
3	160	112	272	20	20
4	160	107	267	20	20
5	160	112	272	20	20

The **Table 3** shows the distribution of instances by class—green and ripe fruit in the training, validation, and test subsets for each fold. The training subset shows the class instances from the original images, where the number of green fruit instances ranges from 870 to 961 per fold, while those of ripe fruit range from 163 to 191. To mitigate the imbalance, the number of green fruit instances was reduced to between 452 and 705 instances per fold, and data augmentation was applied to the minority class (ripe fruit), resulting in increases ranging from 467 to 527 instances. Additionally, the validation and test subsets were composed of instances from the original images.

**Table 3:** Distribution of instances by class in the training, validation, and test sets.

Fold	Training				Validation		Test	
	Green fruit		Ripe fruit		Green	Ripe	Green	Ripe
	Original	Reduced	Original	Increase				
1	870	603	163	503	98	26	172	32
2	903	452	165	467	136	23	101	33
3	941	647	191	527	99	12	100	18
4	961	705	186	479	71	24	108	11
5	885	523	179	513	97	17	158	25

The results for the 100-epoch configuration are presented in **Appendix 1**, while those for the 200-epoch configuration are included in **Appendix 2** (**Tables 8**–**13**). The models, trained independently for each YOLO11 architecture, were evaluated using the five partitions of the test subset. Therefore, the mean and standard deviation presented here summarize the variability associated with model training and the five independent test partitions (one per fold), which correspond to the 200 images in the entire dataset, with no overlap between folds (Hastie et al., 2009; Kohavi, 1995).

**Table 4** summarizes the models’ training performane after 300 epochs for the detection of green and ripe fruits of *Solanum betaceum* Cav. YOLO11m achieved the best overall performance by combining the largest netork capacity with competitive performance on the detection metrics. For the green fruit class, it achieved a precision of 0.9937 ± 0.0026, a recall of 0.9886 ± 0.0062, an mAP50 of 0.9602 ± 0.0120, and an mAP50–95 of 0.9289 ± 0.0188. For ripe fruits, YOLO11m achieved a precision of 0.9985 ± 0.0011, a recall of 0.9967 ± 0.0010, an mAP50 of 0.9909 ± 0.0088, and an mAP50–95 of 0.9839 ± 0.0095, demonstrating very stable performance across the five folds.

**Table 4:** Training results of YOLO11n, YOLO11s, and YOLO11m after 300 epochs.

Model	Class	Precision	Recall	mAP50	mAP50-95	GFLOPs	Parameters (M)
YOLO11n	Green fruit	0.9918 ± 0.0035	0.9516 ± 0.0274	0.9625 ± 0.0222	0.8755 ± 0.0290	6.6	2.59
	Ripe fruit	0.9985 ± 0.0004	0.9905 ± 0.0030	0.9951 ± 0.0003	0.9724 ± 0.0032		
YOLO11s	Green fruit	0.9943 ± 0.0053	0.9706 ± 0.0224	0.9629 ± 0.0164	0.9083 ± 0.0260	21.7	9.43
	Ripe fruit	0.9981 ± 0.0016	0.9961 ± 0.0012	0.9937 ± 0.0023	0.9840 ± 0.0017		
YOLO11m	Green fruit	0.9937 ± 0.0026	0.9886 ± 0.0062	0.9602 ± 0.0120	0.9289 ± 0.0188	68.5	20.05
	Ripe fruit	0.9985 ± 0.0011	0.9967 ± 0.0010	0.9909 ± 0.0088	0.9839 ± 0.0095		

The validation results are shown in **Table 5**. The three YOLO11 variants demonstrated adequate generalization performance on images not used during model training. YOLO11m achieved the best overall balance between the two classes, with a precision of 0.9947 ± 0.0057, a recall of 0.9880 ± 0.0090, an mAP50 of 0.9611 ± 0.0284 and an mAP50–95 of 0.9299 ± 0.0361 for green fruit, and a precision of 0.9975 ± 0.0021, a recall of 0.9964 ± 0.0032, an mAP50 of 0.9958 ± 0.0010, and an mAP50–95 of 0.9857 ± 0.0061 for ripe fruits, representing the best localization performance among the evaluated architectures. YOLO11s exhibited competitive results for ripe fruits, with an mAP50–95 of 0.9827 ± 0.0071, but showed a relative decline for green fruits (mAP50-95: 0.9177 ± 0.0513), confirming the greater visual complexity of this class across all models. YOLO11n, the lightest architecture with 2.59 M parameters and 6.6 GFLOPs, recorded the lowest validation performance for green fruit, with a recall of 0.9585 ± 0.0267 and an mAP50–95 of 0.8758 ± 0.0562, indicating a reduced ability to reliably detect this class, attributable to its lower representational capacity compared to more complex architectures.

**Table 5:** Validation results of YOLO11n, YOLO11s, and YOLO11m after 300 epochs.

Model	Class	Precision	Recall	mAP50	mAP50-95	GFLOPs	Parameters (M)
YOLO11n	Green fruit	0.9881 ± 0.0146	0.9585 ± 0.0267	0.9517 ± 0.0355	0.8758 ± 0.0562	6.6	2.59
	Ripe fruit	0.9968 ± 0.0053	0.9916 ± 0.0050	0.9897 ± 0.0094	0.9710 ± 0.0100		
YOLO11s	Green fruit	0.9917 ± 0.0076	0.9643 ± 0.0219	0.9589 ± 0.0271	0.9177 ± 0.0513	21.7	9.43
	Ripe fruit	0.9978 ± 0.0015	0.9949 ± 00046	0.9945 ± 0.0022	0.9827 ± 0.0071		
YOLO11m	Green fruit	0.9947 ± 0.0057	0.9880 ± 0.0090	0.9611 ± 0.0284	0.9299 ± 0.0361	68.5	20.05
	Ripe fruit	0.9975 ± 0.0021	0.9964 ± 0.0032	0.9958 ± 0.0010	0.9857 ± 0.0061		

The test results are shown in **Table 6**; all three models demonstrated satisfactory generalization performance on images not used during training or validation. YOLO11m, the architecture with the highest representational capacity—with 20.05 M parameters and 68.5 GFLOPs—achieved the highest mAP50–95 for both classes (0.9253 ± 0.0169 for green fruit and 0.9804 ± 0.0079 for ripe fruit), with a precision of 0.9878 ± 0.0055, a recall of 0.9750 ± 0.0148, and a mAP50 of 0.9618 ± 0.0188 for green fruit, and a precision of 0.9950 ± 0.0041, a recall of 0.9958 ± 0.0039, and an mAP50 of 0.9920 ± 0.0047 for ripe fruits, establishing itself as the best-performing localization architecture among those evaluated, although with no statistically significant difference compared to YOLO11s on any metric after Bonferroni correction (**Table 7**). YOLO11s demonstrated competitive results compared to YOLO11m, achieving an mAP50–95 of 0.9034 ± 0.0373 for green fruit and 0.9754 ± 0.0081 for ripe fruit, with a significantly lower computational cost (9.43 M parameters, 21.7 GFLOPs), which positions it as an efficient alternative for resource-constrained environments. YOLO11n, the lightest architecture with 2.59 M parameters and 6.6 GFLOPs, had the lowest mAP50–95 for green fruits (0.8557 ± 0.0611), although it maintained acceptable performance for ripe fruits (0.9642 ± 0.0067), reflecting the limitations of its lower representational capacity for detecting objects with greater visual variability.

**Table 6:** Test results of YOLO11n, YOLO11s, and YOLO11m after 300 epochs.

Model	Class	Precision	Recall	mAP50	mAP50-95	GFLOPs	Parameters (M)
YOLO11n	Green fruit	0.9761 ± 0.0177	0.9428 ± 0.0280	0.9391 ± 0.0397	0.8557 ± 0.0611	6.6	2.59
	Ripe fruit	0.9894 ± 0.0080	0.9885 ± 0.0057	0.9884 ± 0.0067	0.9642 ± 0.0067		
YOLO11s	Green fruit	0.9852 ± 0.0068	0.9586 ± 0.0274	0.9452 ± 0.0365	0.9034 ± 0.0373	21.7	9.43
	Ripe fruit	0.9931 ± 0.0054	0.9932 ± 0.0068	0.9894 ± 0.0070	0.9754 ± 0.0081		
YOLO11m	Green fruit	0.9878 ± 0.0055	0.9750 ± 0.0148	0.9618 ± 0.0188	0.9253 ± 0.0169	68.5	20.05
	Ripe fruit	0.9950 ± 0.0041	0.9958 ± 0.0039	0.9920 ± 0.0047	0.9804 ± 0.0079		

**Table 7:** *Post-hoc* pairwise comparisons and effect sizes (Cohen’s δ) from the repeated-measures linear model with Fold as a fixed blocking factor (*K* = 5, test split, 300 epochs).

Class	Metric	YOLO11n vs. YOLO11s		YOLO11n vs. YOLO11m		YOLO11s vs. YOLO11m	
		$\rho_{adj}$	Cohen’s δ [95% CI]	$\rho_{adj}$	Cohen’s δ [95% CI]	$\rho_{adj}$	Cohen’s δ [95% CI]
Green fruit	Precision	0.416 ns	+0.59 [+0.49, +4.15]	0.203 ns	+0.80 [+0.69, +12.66]	1.000 ns	+1.00 [+0.61, +2.50]
	Recall	0.557 ns	+0.76 [+0.56, +2.21]	0.054 ns	+1.19 [+0.78, +3.09]	0.507 ns	+0.66 [+0.61, +6.45]
	mAP50	1.000 ns	+1.37 [+1.02, +3.80]	0.084 ns	+0.94 [+0.69, +6.21]	0.257 ns	+0.76 [+0.54, +2.10]
	mAP50-95	0.042 *	+1.71 [+1.38, +6.41]	0.006 **	+1.48 [+1.22, +6.11]	0.571 ns	+0.96 [+0.70, +3.15]
Ripe fruit	Precision	0.106 ns	+1.04 [+0.75, +4.75]	0.016 *	+1.39 [+0.83, +4.37]	0.712 ns	+0.99 [+0.57, +2.52]
	Recall	0.030 *	+1.65 [+1.07, +3.79]	0.002 **	+2.96 [+2.18, +7.37]	0.287 ns	+0.68 [+0.56, +3.47]
	mAP50	1.000 ns	+1.77 [+0.86, +6.87]	0.143 ns	+0.91 [+0.76, +4.34]	0.381 ns	+0.59 [+0.51, +2.44]
	mAP50-95	0.001 **	+2.83 [+1.81, +8.95]	*<* 0.001 ***	+3.02 [+2.09, +8.15]	0.093 ns	+1.54 [+1.08, +4.45]
Overall	Precision	0.095 ns	+0.68 [+0.57, +3.39]	0.016 *	+0.94 [+0.76, +14.81]	1.000 ns	+1.04 [+0.61, +2.97]
	Recall	0.524 ns	+1.07 [+0.86, +5.60]	0.038 *	+1.45 [+1.07, +3.55]	0.609 ns	+0.79 [+0.66, +8.37]
	mAP50	1.000 ns	+1.59 [+1.17, +3.67]	0.272 ns	+0.95 [+0.72, +13.50]	0.629 ns	+0.74 [+0.54, +2.24]
	mAP50-95	0.055 ns	+2.40 [+1.96, +9.15]	0.004 **	+2.00 [+1.62, +8.99]	0.776 ns	+1.25 [+0.98, +8.79]

Model: ${Metric}_{ijk}=\beta_{0}+\beta_{1}+\beta_{2}+\gamma_{i}+\epsilon_{ijk}$ with Fold as a fixed blocking factor. Bonferroni correction (*×*3) applied within each row. Cohen’s δ = mean paired difference/SD of paired differences; CI obtained by percentile bootstrap (*B* = 10,000). *ρ<0.05; **ρ<0.01; ***ρ<0.001; ns, not significant.

**Figure 4:**
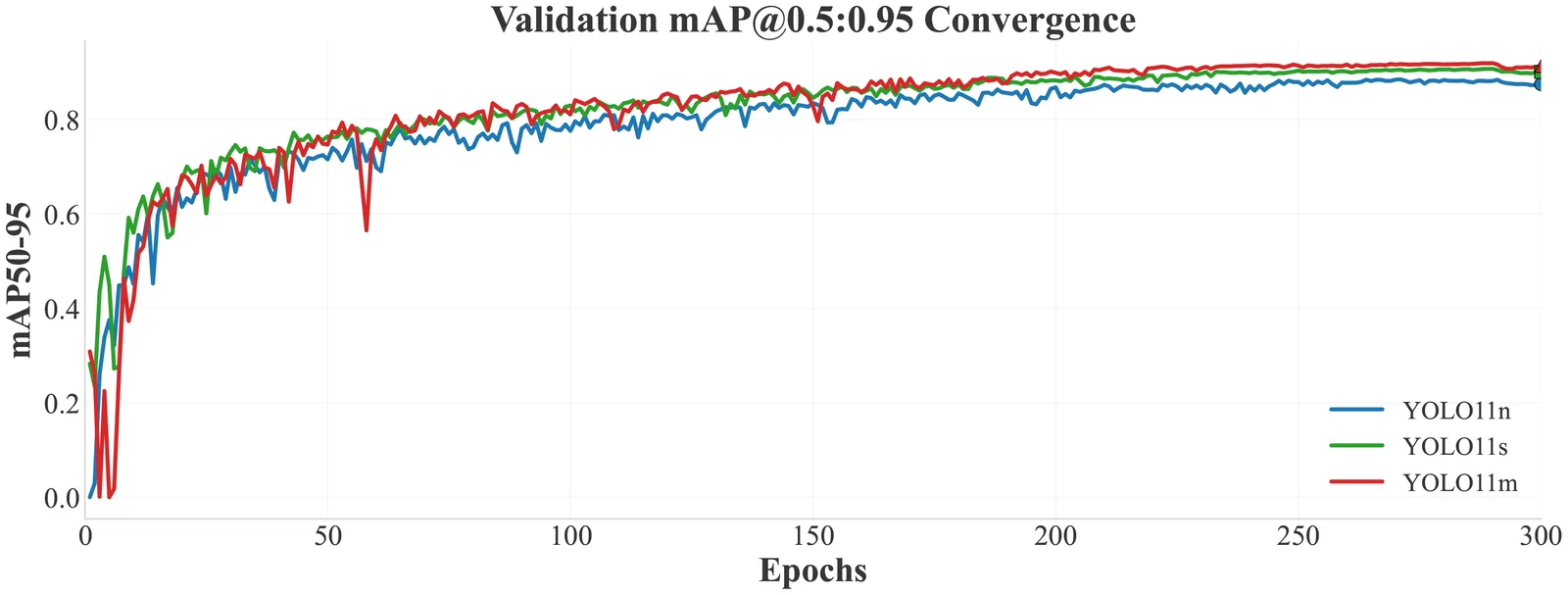
Convergence of the YOLO11 models during training for 300 epochs (Fold 1).

**Figure 4** shows the convergence behavior of the YOLO11n, YOLO11s, and YOLO11m models over 300 training epochs. The validation mAP50–95 curves (fold 1) show a sharp increase during the first 50 epochs, after which the rate of improvement gradually decreases between epochs 180 and 200. Among the evaluated models, YOLO11m consistently achieved the highest validation mAP50–95 throughout the entire training process, closely followed by YOLO11s, while YOLO11n converged to slightly lower values.

**Figure 5:**
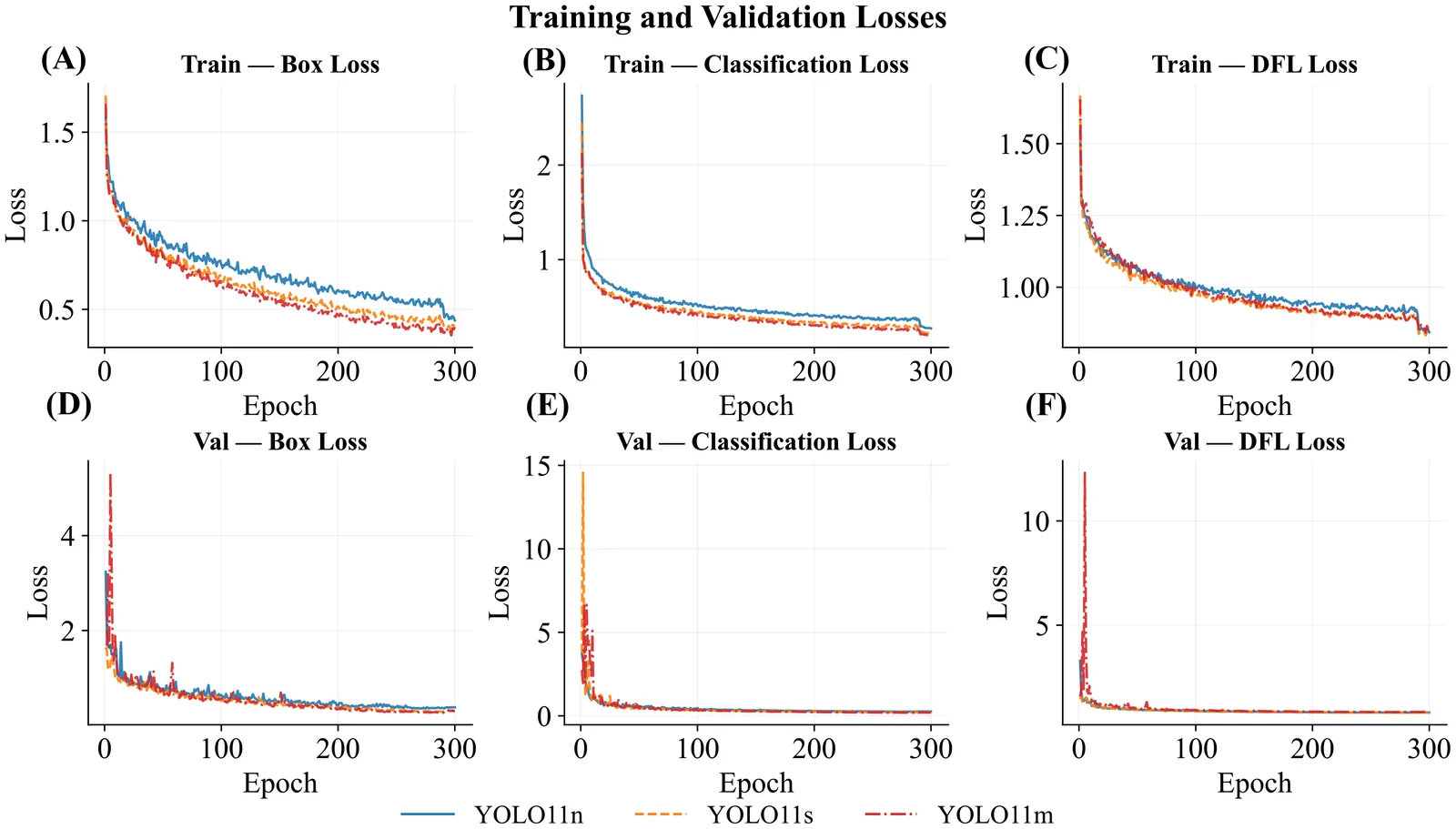
Loss curves during training and validation for 300 epochs (Fold 1). **(A)** Training box loss; **(B)** training classification loss; **(C)** training DFL loss; **(D)** validation box loss; **(E)** validation classification loss; and **(F)** validation DFL loss.

**Figure 5** shows the evolution of the training and validation losses for the three variants of YOLO11 over 300 training epochs. All models exhibited a rapid decrease in Box Loss, Classification Loss, and Distribution Focal Loss during the first few epochs, followed by a gradual stabilization, indicating satisfactory convergence. The similarity between the training and validation curves throughout the optimization process demonstrates stable learning without significant divergences. Although YOLO11m exhibited slightly greater fluctuations during the initial validation phase, these quickly disappeared, and the model converged to stable loss values comparable to those of YOLO11n and YOLO11s. Furthermore, YOLO11s and YOLO11m consistently achieved lower final training losses than YOLO11n, because the larger architectures learned more discriminative feature representations while maintaining stable optimization.

**Figure 6:**
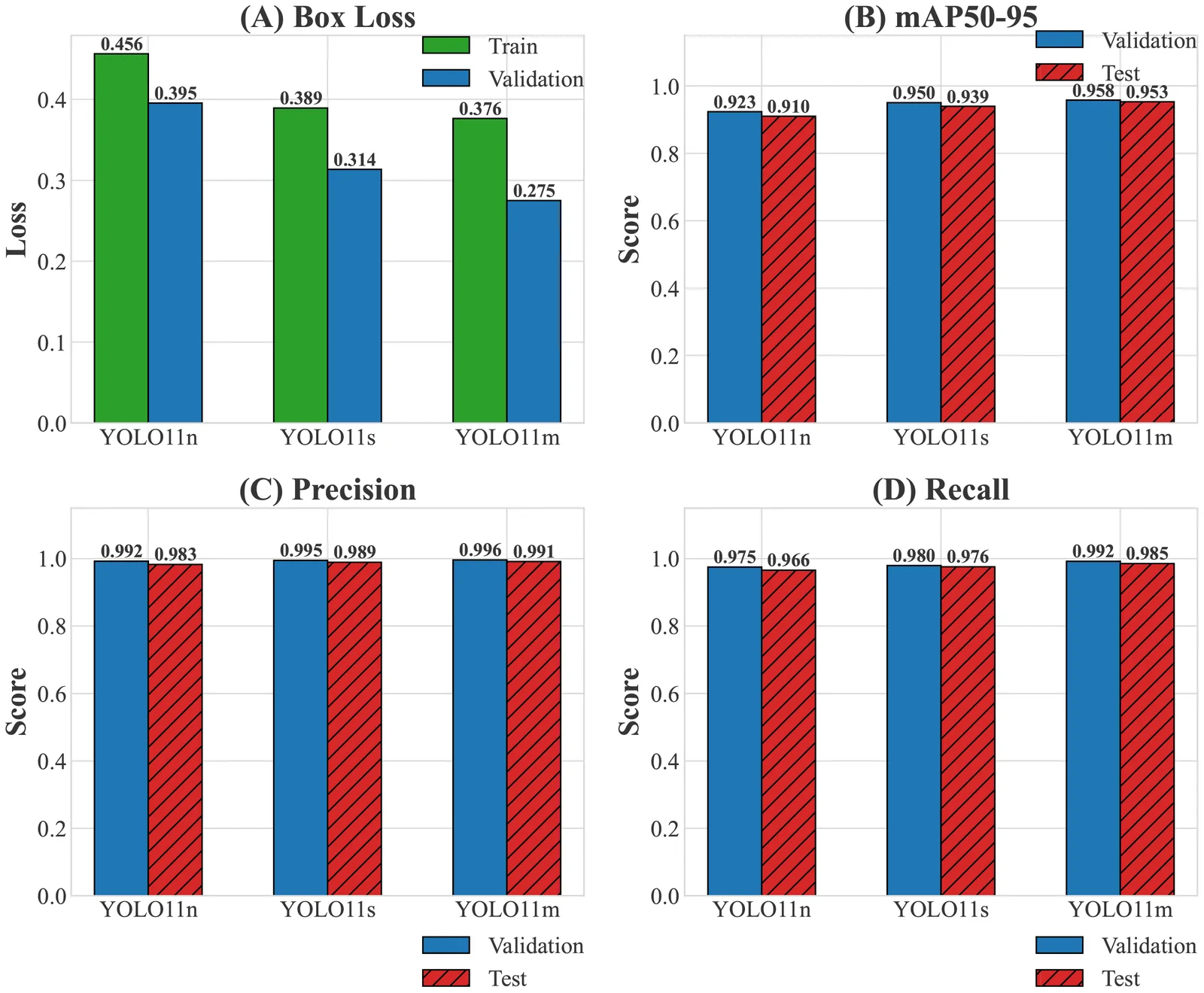
Comparison of box loss, mAP50-95, precision, and recall on the training, validation, and test datasets for the YOLO11n, YOLO11s, and YOLO11m architectures. **(A)** Box loss for the training and validation sets; **(B)** mAP50–95 for the validation and test sets; **(C)** precision for the validation and test sets; and **(D)** recall for the validation and test sets.

**Figure 6** presents a comparison of the performance of the three YOLO11 architectures in terms of training and validation box loss, mAP50-95, precision, and recall (simple average across classes). Box loss shows a gradual decrease as the model’s complexity increases, with training values of 0.456, 0.389, and 0.376 for YOLO11n, YOLO11s, and YOLO11m, respectively, and a similar trend in validation (0.395, 0.314, and 0.275), indicating a reduction in the error associated with bounding box regression in the higher-capacity architectures. Regarding mAP50-95, YOLO11m achieved the highest values in both validation (0.958) and test (0.953), followed by YOLO11s (0.950 and 0.939) and YOLO11n (0.923 and 0.910), respectively, confirming YOLO11m’s superior performance on this more demanding evaluation metric. Precision showed high values across both datasets, with an overall range of 0.983 to 0.996. In validation, the values were 0.992, 0.995, and 0.996 for YOLO11n, YOLO11s, and YOLO11m, respectively, while in the test set, they were 0.983, 0.989, and 0.991.

These results show a slight improvement in precision as the model’s capacity increases. Recall followed a pattern consistent with mAP50-95, with YOLO11m achieving the highest values in validation (0.992) and test (0.985), compared to 0.980 and 0.976 for YOLO11s, and 0.975 and 0.966 for YOLO11n.

### 3.2 Statistical comparison of architectures

To compare the architectures, we used a linear model with Architecture and Fruit Class as fixed effects and Fold as a fixed block factor (repeated-measures design). This approach is preferable to a mixed model with a random interaction when the number of folds is small (*K* = 5), since the variance between folds cannot be reliably estimated with so few levels (Gelman and Hill, 2006). The resulting linear model is presented in Equation 4:

(4) $${\text{Metric}}_{ijk}\;=\;\beta_{0}\;+\;\beta_{1}\;\left({\text{Architecture}}_{j}\right)\;+\;\beta_{2}\left({\text{Class}}_{k}\right)\;+\;{\text{γ}}_{i}\left(\text{Fold}\right)\;+\;\epsilon_{ijk},$$

Where $\gamma_{i}$ are fixed fold-specific deviations. Pairwise *post-hoc* contrasts were evaluated with Bonferroni correction for three comparisons. Because *K* = 5 limits statistical power, we report Cohen’s *d* (paired, mean difference divided by standard deviation of differences) as the primary measure of effect magnitude, alongside Bonferroni-adjusted *p*-values. Analyses were conducted in Python (statsmodels 0.14).

**Table 7** shows the *post hoc* comparisons and Cohen’s *d* effect sizes. YOLO11m consistently achieved the highest mean scores across all metrics. The effect sizes compared to YOLO11n ranged from large to very large (*d* = 0.94–3.02), and the differences reached statistical significance in Precision (ripe fruit, $\rho_{adj}=0.016$), Recall (overall, $\rho_{adj}=0.038$; ripe fruit, $\rho_{adj}=0.002$) and mAP50-95 (green fruit, $\rho_{adj}=0.006$; ripe fruit, $\rho_{adj}<0.001$; overall, $\rho_{adj}=0.004$).

However, no significant differences were detected between YOLO11m and YOLO11s on any metric (all $\rho_{adj}values\mathrm{\&gt;}0.05$), even though the effect sizes ranged from moderate to large ( δ=0.59-1.54). This lack of significance reflects the limited statistical power inherent in the *K* = 5 convolutions, rather than the absence of a practical difference. Similarly, YOLO11s differed significantly from YOLO11n only in mAP50-95 (green fruit, $\rho_{adj}=0.042$; ripe fruit, $\rho_{adj}=0.001$) and in recall (ripe fruit, $\rho_{adj}=0.030$).

### 3.3 Yolo11 model detection

The results obtained are consistent with recent literature, which highlights the effectiveness of YOLO algorithms for fruit detection in agricultural scenarios (Ye et al., 2024). Under real-world field conditions, characterized by variable lighting, partial occlusions, scale differences, and complex vegetation backgrounds, the YOLO11m and YOLO11s architectures demonstrated excellent performance in recognizing green and ripe fruits of *Solanum betaceum* Cav. This performance was more consistent in YOLO11m, which maintained high levels of precision, recall, and mAP on the training, validation, and test datasets, while requiring greater computational complexity. In contrast, YOLO11n did not show an improvement proportional to its reduced size, especially in the detection of green fruit, which is an issue that should be addressed in future model improvements.

**Figure 7:**
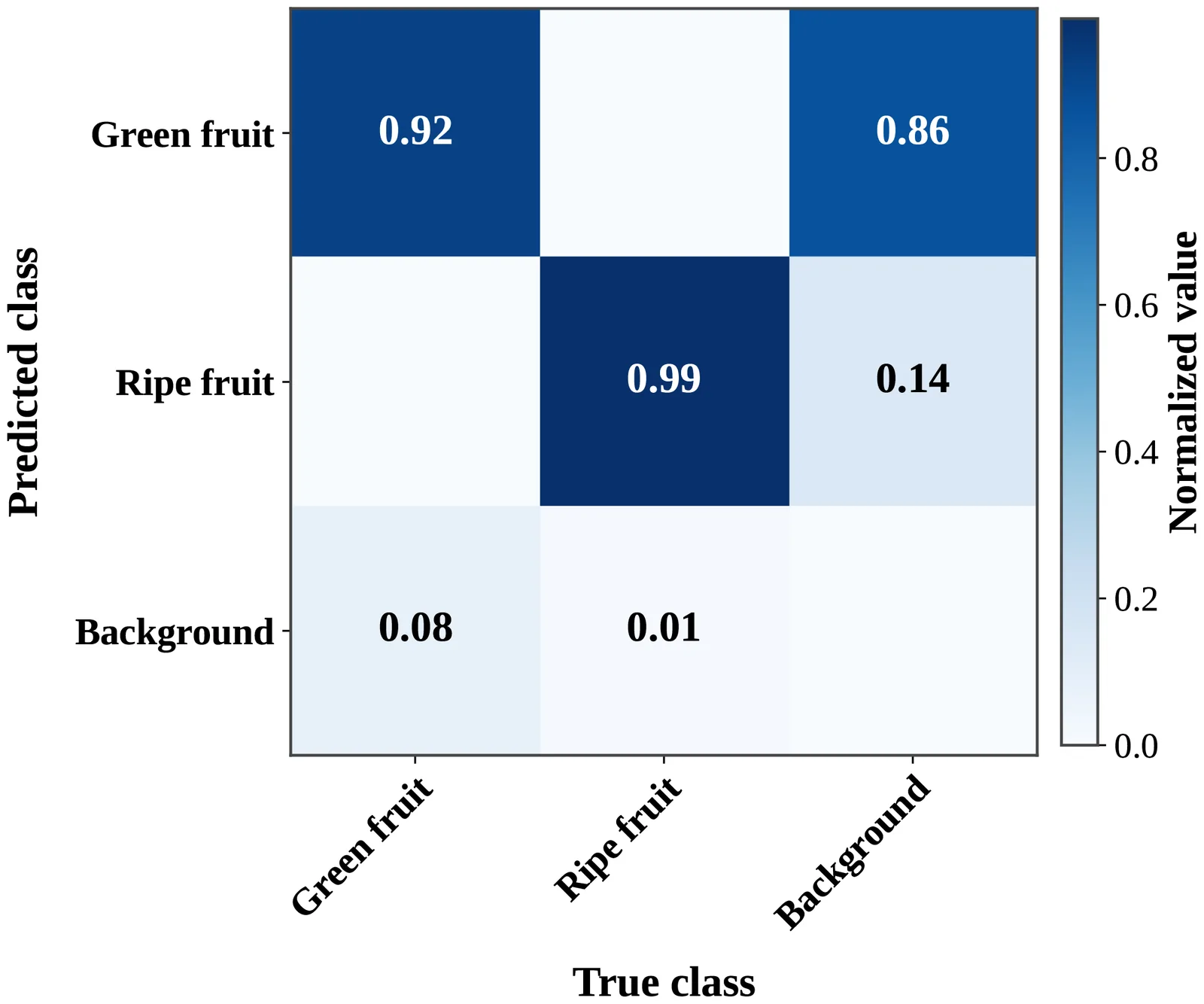
Normalized confusion matrix for the YOLO11m model on the test set (Fold 1).

The normalized confusion matrix shown in **Figure 7** demonstrates varying classification performance across classes (Fold 1). Ripe fruits achieve the highest correct detection rate (99%), with only 1% of instances incorrectly assigned to the background. green fruits reach a correct detection rate of 92%, with 8% of instances misclassified as background, reflecting the greater difficulty in distinguishing this class from the surrounding foliage due to their chromatic and textural similarity. Regarding background regions, 86% were incorrectly predicted as green fruit and 14% as ripe fruit, indicating that the model’s false positive detections are predominantly associated with the green fruit class. It should be noted that these values represent row-normalized proportions of misclassified background instances, not the absolute false positive rate relative to the total number of background regions in the images. Taken together, these results confirm that the discrimination of green fruit from vegetative background constitutes the primary classification challenge for the model under the evaluated field conditions (Lu et al., 2022).

The main qualitative characteristics that make it difficult to detect green fruit stem from their chromatic similarity to the foliage background a form of chromatic camouflage where the color of the green fruit’s skin blends with the hue of the surrounding leaves under natural lighting conditions, drastically reducing the discriminatory power of the color descriptors (Jia et al., 2022; Lu et al., 2022); this chromatic convergence is exacerbated by the textural similarity between the fruit’s surface and the surrounding vegetation, as both share patterns of roughness and surface variability that confuse segmentation methods based exclusively on texture (Wang et al., 2025; Fan et al., 2022). Additionally, fruits in early stages of development exhibit less morphological prominence and less defined edges, which increases the likelihood that they will be visually absorbed by the background and misclassified as foliage (Jia et al., 2022; Huang et al., 2026). Finally, the variability of lighting conditions in field environments enhances or attenuates these similarities in unpredictable ways, rendering traditional color thresholds inadequate for the robust identification of green fruits (Lu et al., 2022; Yu et al., 2025).

**Figure 8:**
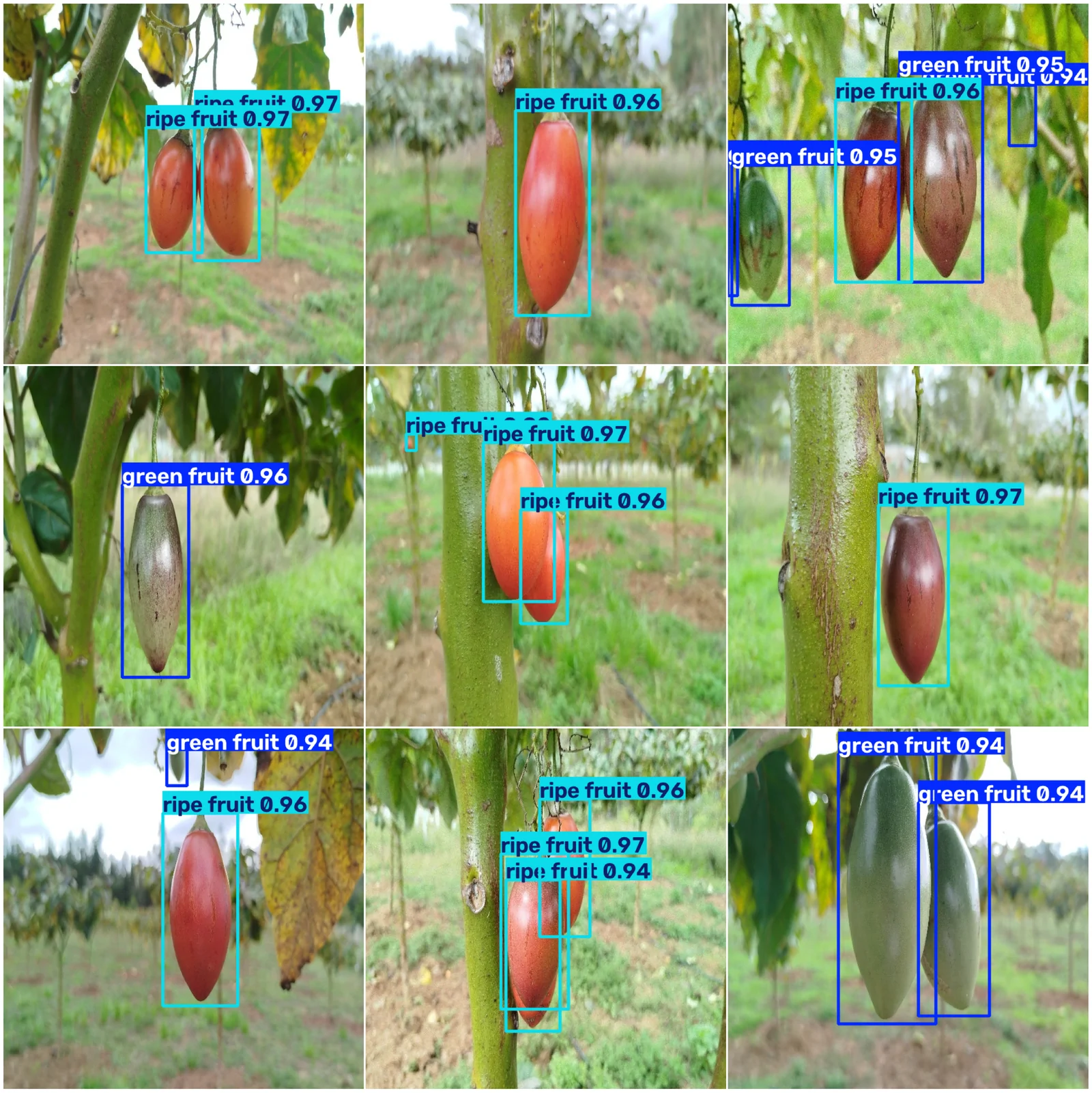
Prediction of *S. betaceum Cav.* fruit maturity using YOLO11m (Fold 1).

**Figure 8** shows the detections made by the YOLO11m model (Fold 1) on images from the test set, illustrating its ability to simultaneously locate and classify green and ripe fruits under real-world field conditions. The generated bounding boxes exhibit consistently high confidence scores, predominantly ranging between 0.94 and 0.97, for both unripe and ripe fruits, reflecting the model’s high certainty in its predictions under various lighting conditions, fruit density, and partial occlusion. The model is observed to maintain robust performance in scenes with high object density, where multiple fruits of both classes coexist in proximity, as well as in the face of variations in the orientation, relative size, and ripeness level of the fruits.

## 4 Discussion

The cross-validation strategy with *K* = 5 folds adopted in this study is based on established practices in machine learning (Kohavi, 1995). However, it is important to recognize that this experimental design imposes inherent constraints on statistical inference. The variance across folds introduces a source of variability: the stochastic data augmentation applied exclusively to the training subset of each fold. As shown in **Table 2**, the number of upsampled images included in the training set ranged from 96 to 113 depending on the fold, resulting in heterogeneous final training set sizes (256 to 273 images). This variability is amplified at the instance level (**Table 3**), where oversampling of the minority class (ripe fruits) through geometric and photometric transformations and Gaussian noise produced differential increases ranging from 467 to 527 additional instances for ripe fruits, and from 452 to 705 for green fruits. Due to the increase in offline data, each fold was trained with a slightly different distribution of the augmentation space. This heterogeneity is reflected in the wider bootstrap confidence intervals, resulting in validation and test metrics that show standard deviations higher than those expected from partitioned sampling alone.

The results obtained demonstrate that models based on the YOLO11 family constitute a technically viable alternative for the automatic detection and visual assessment of the ripeness of *Solanum betaceum* Cav. under real field conditions. This finding is significant given that the tree tomato is a fruit of remarkable nutritional, functional, and economic value, characterized by a phytochemical profile rich in bioactive compounds, vitamins, and minerals, as well as by distinct physiological changes during ripening (MaChado et al., 2024; Rohilla and Mahanta, 2024; Hu et al., 2023; Ma et al., 2025). In conventional agricultural practice, determining the stage of ripeness and making harvest-related decisions relies predominantly on manual visual assessments, which exhibit high inter-observer variability due to the grower’s experience, ambient lighting conditions, accessibility to the fruit, and the architectural complexity of the canopy. In this context, the implementation of computer vision systems addresses a specific need in the agricultural sector by enabling the observation, comparison, and recording of ripeness status in an objective, timely, and reproducible manner (Wei et al., 2024; Xu et al., 2024; Zhu et al., 2025).

A distinctive methodological strength of this study lies in the fact that the dataset was collected using mobile devices in a real agroecological environment, as opposed to highly controlled laboratory settings. Although these factors increase the complexity of automatic detection, they allow the models to be evaluated under conditions that closely approximate those of an actual production application. Previous studies have emphasized that fruit detection in real plantations requires architectures capable of handling occlusions, heterogeneous changes in lighting, object overlap, scale variation, and unstructured backgrounds (Lin et al., 2025; Liu et al., 2025; Wang et al., 2025; Kamat et al., 2025; Tang et al., 2023; Ma et al., 2025).

The results for the 300-epoch configuration, presented in **Tables 4**–**6**, show that YOLO11m consistently achieved the best localization performance across the three evaluated subsets. On the training set, YOLO11m achieved an mAP50–95 of 0.9289 *±* 0.0188 for green fruit and 0.9839 *±* 0.0095 for ripe fruit. In validation, these values rose to 0.9299 *±* 0.0361 and 0.9857 *±* 0.0061, respectively, while on the test set—which is most relevant for evaluating the model’s generalization ability—the values were 0.9253 *±* 0.0169 for green fruits and 0.9804 *±* 0.0079 for ripe fruits.

The hybrid balancing strategy—which involved offline oversampling of the minority class combined with undersampling of the majority class—was applied exclusively to the training subsets of each fold. This prevented data leakage but introduced systematic variability across folds. Although this variability contributes to the reported standard deviations, it also serves as a regularization mechanism that improves the model’s robustness under heterogeneous field conditions. The *online* data augmentation natively implemented by YOLO11 worked synergistically with the *offline* augmentation, processing stochastically distinct variations across epochs. This dual increase explains the rapid convergence observed during the first 50 epochs (**Figure 4**) and the subsequent stabilization of the loss curves (**Figure 5**), particularly in YOLO11m and YOLO11s, whose greater parametric capacity allowed them to capitalize on the artificial diversity of the training dataset without evident overfitting.

YOLO11s established itself as a highly competitive intermediate alternative. On the test dataset, this architecture achieved a *recall* of 0.9586 *±* 0.0274 for green fruit and 0.9932 *±* 0.0068 for ripe fruit, with mAP50 values of 0.9452 *pm*0.0365 and 0.9894 *±* 0.0070, respectively, and mAP50–95 values of 0.9034 *±* 0.0373 and 0.9754 *±* 0.0081. Although these metrics are slightly lower than those obtained by YOLO11m, the magnitude of this difference is relatively modest considering that YOLO11s uses less than half the number of parameters (9.43 M) and approximately one-third of the computational load (21.7 GFLOPs). This balance between detection precision and algorithmic efficiency is particularly relevant for field technical support applications, where processing must be performed on mobile or edge devices with limited memory and processing capacity (Gao et al., 2025; Lawal et al., 2024). In this regard, YOLO11s emerges as a practical option when deployment feasibility is prioritized over maximum absolute precision.

For its part, YOLO11n, the most compact architecture (2.59 M parameters, 6.6 GFLOPs), maintained acceptable performance for the ripe fruit class, with an mAP50–95 of 0.9642 *±* 0.0067 on the test set, close to that of the more complex architectures. However, its performance in detecting green fruit was lower, with an mAP50–95 of 0.8557 *±* 0.0611, a *recall* of 0.9428 *±* 0.0280, and a precision of 0.9761 *±* 0.0177. This limitation is due to the network’s reduced representational capacity to learn discriminative visual patterns of objects whose morphology—green hues, smooth texture, and oval shape—exhibits high chromatic and structural similarity to the vegetative background composed of leaves, branches, and shaded areas. This finding is consistent with the literature, which indicates that detecting green fruit against chromatically similar backgrounds is one of the most complex challenges in uncontrolled agricultural settings (Chen et al., 2025; Hao et al., 2025; Lin et al., 2024; Tang et al., 2025; Zhang et al., 2025). It is worth noting that the higher standard deviation observed in YOLO11n for green fruits (*± *0.0611 in mAP50-95) reflects not only the intrinsic difficulty of the class but also greater sensitivity to variability in data augmentation across folds, given that its limited parametric capacity prevents it from generalizing effectively in the face of the diversity of applied transformations.

In agricultural regions such as the Peruvian Amazon, where tree tomatoes represent an agricultural alternative of growing economic importance, a tool based on images captured using mobile devices could facilitate technical assistance, optimize harvest planning, and reduce systematic errors associated with subjective visual assessment. The goal of this technology is not to replace farmers’ empirical knowledge, but rather to complement it with an objective tool that allows for the observation, comparison, and recording of the phenological status of the fruits in a standardized manner. This approach aligns with the principles of precision agriculture, where artificial intelligence progressively adapts to specific field challenges, moving beyond highly controlled experimental settings (Deng et al., 2025; Pugazhendi et al., 2026).

However, the results must be interpreted in light of certain methodological limitations. The dataset was collected at a single experimental station during a specific sampling period (February through May 2025); therefore, it is not possible to guarantee that the model would perform equally well in orchards at different elevations, with different climatic conditions, soil conditions, or agronomic management systems. Furthermore, fruit maturity was classified into a binary taxonomy of “green fruit” and “ripe fruit” using external color as the primary criterion. Although this methodological decision simplified the annotation protocol and reduced the incidence of inter-observer classification errors, the commercial maturity of *S. betaceum* Cav. is a gradual process that includes intermediate stages relevant to harvest and postharvest logistics.

Consequently, future research should focus on expanding and balancing the image dataset, incorporating intermediate maturity stages, and conducting external validation of the models in production settings with heterogeneous capture conditions to rigorously evaluate their transferability to real-world agroecological contexts. From an architectural perspective, the incorporation of attention mechanisms, multiscale fusion, instance segmentation, and hybrid architectures based on *Transformers* represents a promising line of development for improving object discrimination under conditions of high visual variability. With these improvements, YOLO11s and, eventually, YOLO11n could establish themselves as practical support tools for producers, researchers, and agricultural extension services involved with Andean and tropical crops.

## 5 Conclusions

The three YOLO11 variants evaluated demonstrate that lightweight object detection architectures constitute a technically viable pathway for the automatic assessment of *Solanum betaceum* Cav. maturity under real field conditions, offering an objective alternative to manual visual inspection.

YOLO11m achieved the strongest overall localization performance, recording the highest mean mAP50–95 across all evaluation subsets, with large effect sizes relative to YOLO11n ( δ=0.94-3.02). On the test set, it reached an mAP50 of 0.9618 *±* 0.0188 for green fruit and 0.9920 *±* 0.0047 for ripe fruit, and an mAP50–95 of 0.9253 *±* 0.0169 and 0.9804 *±* 0.0079, respectively. This performance is attributable to its greater representational capacity (20.05 M parameters, 68.5 GFLOPs), which allowed the model to learn more discriminative features across heterogeneous field conditions. However, its advantage over YOLO11s did not reach statistical significance on any metric ( $\rho_{adj}\mathrm{\&gt;}0.05$; **Table 7**), indicating that the performance gap between the two architectures is practically meaningful but not conclusively established at *K* = 5.

YOLO11s emerged as the most practically balanced architecture. It achieved an mAP50–95 of 0.9034 *±* 0.0373 for green fruit and 0.9754 *±* 0.0081 for ripe fruit on the test set, at less than half the parameter count (9.43 M) and approximately one-third of the computational load (21.7 GFLOPs) of YOLO11m. This efficiency–accuracy trade-off positions YOLO11s as the preferred candidate for deployment on mobile or edge devices in field technical assistance applications.

YOLO11n, the most compact architecture (2.59 M parameters, 6.6 GFLOPs), maintained acceptable performance for ripe fruit (mAP50-95: 0.9642 *±* 0.0067) but showed substantially lower and more variable results for green fruit (mAP50-95: 0.8557 *±* 0.0611; recall: 0.9428 *±* 0.0280). This limitation reflects its reduced capacity to resolve the chromatic and textural overlap between unripe fruit and the vegetative background, a challenge that is exacerbated when training distributions vary across folds.

Across all architectures, ripe fruit was consistently the easier class to detect, owing to the strong visual contrast of its orange, reddish, or purple coloration against the canopy. Green fruit remained the primary discrimination challenge, sharing hue, texture, and morphology with leaves, branches, and shaded regions a difficulty that no model fully resolved and that future work should specifically address.

These findings represent an initial but meaningful validation of YOLO11-based maturity detection for tree tomato production. Their scope is bounded by the use of a single experimental station, a four month collection window, and a binary maturity taxonomy. To strengthen transferability and operational applicability, future research should expand the dataset across orchards, altitudes, and seasons; incorporate intermediate ripeness stages; and integrate physicochemical quality indicators such as soluble solids, acidity, firmness, and pigmentation. With those extensions, YOLO11s and potentially YOLO11n could become practical decision-support tools for producers, agronomists, and extension services working with Andean and tropical fruit systems.

## Data Availability

The datasets presented in this study can be found in online repositories. The names of the repository/repositories and accession number(s) can be found below: https://github.com/omercruz-sys/YOLO11---Solanum-betaceum-Cav- and https://doi.org/10.5281/zenodo.21167173.

## Appendix 1

**Table 8:** Training results for YOLO11n, YOLO11s, and YOLO11m at 100 epochs.

Model	Class	Precision	Recall	mAP50	mAP50-95	GFLOPs	Parameters (M)
YOLO11n	Green fruit	0.9786 ± 0.0054	0.9147 ± 0.0395	0.9468 ± 0.0292	0.8171 ± 0.0388	6.6	2.59
	Ripe fruit	0.9896 ± 0.0059	0.9803 ± 0.0080	0.9824 ± 0.0066	0.9200 ± 0.0086		
YOLO11s	Green fruit	0.9816 ± 0.0031	0.9175 ± 0.0360	0.9472 ± 0.0230	0.8476 ± 0.0339	21.7	9.43
	Ripe fruit	0.9903 ± 0.0072	0.9760 ± 0.0111	0.9859 ± 0.0089	0.9446 ± 0.0082		
YOLO11m	Green fruit	0.9771 ± 0.0168	0.9334 ± 0.0241	0.9501 ± 0.0158	0.8763 ± 0.0352	68.5	20.05
	Ripe fruit	0.9984 ± 0.0015	0.9918 ± 0.0039	0.9912 ± 0.0089	0.9595 ± 0.0046		

**Table 9:** Validation results for YOLO11n, YOLO11s, and YOLO11m at 100 epochs.

Model	Class	Precision	Recall	mAP50	mAP50-95	GFLOPs	Parameters (M)
YOLO11n	Green fruit	0.9671 ± 0.0107	0.8984 ± 0.0608	0.9299 ± 0.0422	0.8026 ± 0.0655	6.6	2.59
	Ripe fruit	0.9874 ± 0.0014	0.9806 ± 0.0027	0.9805 ± 0.0043	0.9100 ± 0.0145		
YOLO11s	Green fruit	0.9863 ± 0.0127	0.9171 ± 0.0417	0.9489 ± 0.0348	0.8568 ± 0.0580	21.7	9.43
	Ripe fruit	0.9955 ± 0.0050	0.9683 ± 0.0145	0.9880 ± 0.0067	0.9465 ± 0.0103		
YOLO11m	Green fruit	0.9874 ± 0.0057	0.9691 ± 0.0222	0.9411 ± 0.0275	0.8828 ± 0.0590	68.5	20.05
	Ripe fruit	0.9962 ± 0.0032	0.9938 ± 0.0058	0.9904 ± 0.0068	0.9579 ± 0.0097		

**Table 10:** Test results for YOLO11n, YOLO11s, and YOLO11m at 100 epochs.

Model	Class	Precision	Recall	mAP50	mAP50-95	GFLOPs	Parameters (M)
YOLO11n	Green fruit	0.9525 ± 0.0034	0.8708 ± 0.0595	0.9064 ± 0.0048	0.7738 ± 0.0543	6.6	2.59
	Ripe fruit	0.9691 ± 0.0061	0.9545 ± 0.0044	0.9570 ± 0.0056	0.9055 ± 0.0133		
YOLO11s	Green fruit	0.9664 ± 0.0063	0.9152 ± 0.0377	0.9176 ± 0.0067	0.8421 ± 0.0496	21.7	9.43
	Ripe fruit	0.9877 ± 0.0039	0.9653 ± 0.0039	0.9827 ± 0.0022	0.9397 ± 0.0069		
YOLO11m	Green fruit	0.9803 ± 0.0064	0.9580 ± 0.0265	0.9250 ± 0.0083	0.8709 ± 0.0378	68.5	20.05
	Ripe fruit	0.9920 ± 0.0052	0.9900 ± 0.0053	0.9860 ± 0.0051	0.9481 ± 0.0080		

## Appendix 2

**Table 11:** Training results for YOLO11n, YOLO11s, and YOLO11m at 200 epochs.

Model	Class	Precision	Recall	mAP50	mAP50-95	GFLOPs	Parameters (M)
YOLO11n	Green fruit	0.9878 ± 0.0049	0.9448 ± 0.0252	0.9330 ± 0.0223	0.8632 ± 0.0247	6.6	2.59
	Ripe fruit	0.9951 ± 0.0048	0.9857 ± 0.0080	0.9883 ± 0.0060	0.9735 ± 0.0060		
YOLO11s	Green fruit	0.9875 ± 0.0061	0.9603 ± 0.0249	0.9411 ± 0.0210	0.8730 ± 0.0229	21.7	9.43
	Ripe fruit	0.9939 ± 0.0049	0.9840 ± 0.0090	0.9897 ± 0.0068	0.9610 ± 0.0335		
YOLO11m	Green fruit	0.9803 ± 0.0152	0.9701 ± 0.0207	0.9491 ± 0.0202	0.8830 ± 0.0227	68.5	20.05
	Ripe fruit	0.9992 ± 0.0004	0.9963 ± 0.0040	0.9958 ± 0.0048	0.9804 ± 0.0017		

**Table 12:** Validation results for YOLO11n, YOLO11s, and YOLO11m at 200 epochs.

Model	Class	Precision	Recall	mAP50	mAP50-95	GFLOPs	Parameters (M)
YOLO11n	Green fruit	0.9546 ± 0.0245	0.9311 ± 0.0208	0.9193 ± 0.0202	0.8509 ± 0.0230	6.6	2.59
	Ripe fruit	0.9920 ± 0.0095	0.9435 ± 0.0224	0.9842 ± 0.0076	0.9473 ± 0.0301		
YOLO11s	Green fruit	0.9536 ± 0.0149	0.9336 ± 0.0177	0.9278 ± 0.0194	0.8600 ± 0.0222	21.7	9.43
	Ripe fruit	0.9949 ± 0.0051	0.9417 ± 0.0090	0.9919 ± 0.0045	0.9777 ± 0.0020		
YOLO11m	Green fruit	0.9850 ± 0.0153	0.9671 ± 0.0277	0.9357 ± 0.0185	0.8705 ± 0.0222	68.5	20.05
	Ripe fruit	0.9965 ± 0.0042	0.9812 ± 0.0353	0.9936 ± 0.0048	0.9652 ± 0.0316		

**Table 13:** Test results for YOLO11n, YOLO11s, and YOLO11m at 200 epochs.

Model	Class	Precision	Recall	mAP50	mAP50-95	GFLOPs	Parameters (M)
YOLO11n	Green fruit	0.9529 ± 0.0225	0.9310 ± 0.0285	0.9278 ± 0.0108	0.8362 ± 0.0230	6.6	2.59
	Ripe fruit	0.9855 ± 0.0083	0.9554 ± 0.0106	0.9700 ± 0.0099	0.9595 ± 0.0085		
YOLO11s	Green fruit	0.9703 ± 0.0222	0.9493 ± 0.0204	0.9397 ± 0.0106	0.8458 ± 0.0212	21.7	9.43
	Ripe fruit	0.9888 ± 0.0052	0.9709 ± 0.0049	0.9834 ± 0.0035	0.9716 ± 0.0059		
YOLO11m	Green fruit	0.9814 ± 0.0135	0.9730 ± 0.0148	0.9574 ± 0.0147	0.8905 ± 0.0162	68.5	20.05
	Ripe fruit	0.9949 ± 0.0044	0.9929 ± 0.0041	0.9912 ± 0.0040	0.9776 ± 0.0018		

## References

[B1] Ajil A. Ali A. Kiran Reddy A. K. Vasista C Reddy L. Guna Sekhar Reddy A. V. Goud O. (2023). Fruit ripeness assertion using deep learning. Int. J. Hum. Comput. Intell. 2 (2), 63–72. doi: 10.5281/ZENODO.7900479

[B2] Ali M. L. Zhang Z. (2024). The YOLO framework: A comprehensive review of evolution, applications, and benchmarks in object detection. Computers 13, 336. doi: 10.3390/computers1312033630654563

[B3] Alzubaidi L. Zhang J. Humaidi A. J. Al-Dujaili A. Duan Y. Al-Shamma O. . (2021). Review of deep learning: Concepts, CNN architectures, challenges, applications, future directions. J. Big Data 8, 53. doi: 10.1186/s40537-021-00444-8 33816053 PMC8010506

[B4] Anjali Jena A. Bamola A. Mishra S. Jain I. Pathak N. . (2024). State-of-the-art non-destructive approaches for maturity index determination in fruits and vegetables: Principles, applications, and future directions. Food Prod. Process. Nutr. 6, 56. doi: 10.1186/s43014-023-00205-538164791

[B67] Anuyah S. Singh M. K. Nyavor H. (2024). Advancing clinical trial outcomes using deep learning and predictive modelling: Bridging precision medicine and patient-centered care. World J. Advanced Res. Rev. 24, 001–025. doi: 10.30574/wjarr.2024.24.3.3671

[B5] Astiti Asih I. A. R. Manuaba I. B. P. Berata K. Rita W. S. Suirta W. (2023). “ *In vivo* evaluation of antioxidant activity of flavonoid glycoside extract fromtamarillo (*Solanum betaceum* Cav)”, in: IOP Conference Series: Earth and Environmental Science ( IOP Publishing Ltd), 1177. 012039. doi: 10.1088/1755-1315/1177/1/012039

[B6] Bochkovskiy A. Wang C.-Y. Liao H.-Y. M. (2020). YOLOv4: Optimal speed and accuracy of object detection. arXiv, 1–17. doi: 10.48550/ARXIV.2004.10934

[B7] Buslaev A. Iglovikov V. I. Khvedchenya E. Parinov A. Druzhinin M. Kalinin A. A. (2020). Albumentations: Fast and flexible image augmentations. Information 11, 125. doi: 10.3390/info1102012530654563

[B8] Caeiro A. Correia S. Canhoto J. (2023). Influence of flavonoids and phenolic acids in the *in vitro* propagation of tamarillo shoots. Acta Hortic., 189–196. doi: 10.17660/ActaHortic.2023.1359.24

[B9] Chen B.-J. Bu J.-Y. Xia J.-L. Li M.-X. Su W.-H. (2025). AFBF-YOLO: An improved YOLO11n algorithm for detecting bunch and maturity of cherry tomatoes in greenhouse environments. Plants 14, 2587. doi: 10.3390/plants14162587 40872209 PMC12389433

[B10] Chen J. Wu J. Wang Z. Qiang H. Cai G. Tan C. . (2021). Detecting ripe fruits under natural occlusion and illumination conditions. Comput. Electron. Agric. 190, 106450. doi: 10.1016/j.compag.2021.10645042574925

[B11] Ching T. Himmelstein D. S. Beaulieu-Jones B. K. Kalinin A. A. Do B. T. Way G. P. . (2018). Opportunities and obstacles for deep learning in biology and medicine. J. R. Soc Interface 15, 20170387. doi: 10.1098/rsif.2017.0387 29618526 PMC5938574

[B12] Chunnapiya P. Visutsak P. (2025). Enhancing automatic electric vehicle charging: A deep learning approach with YOLO and feature extraction techniques. Front. Comput. Sci. 7, 1505446. doi: 10.3389/fcomp.2025.1505446

[B13] Cong X. Li S. Chen F. Liu C. Meng Y. (2023). A review of YOLO object detection algorithms based on deep learning. Front. Computing Intelligent Syst. 4, 17–20. doi: 10.54097/fcis.v4i2.9730

[B14] Coyago-Cruz E. Guachamin A. Méndez G. Moya M. Martínez A. Viera W. . (2023). Functional and antioxidant evaluation of two ecotypes of control and grafted tree tomato (*Solanum betaceum*) at different altitudes. Foods 12, 3494. doi: 10.3390/foods12183494 37761202 PMC10530088

[B15] Cuong N. H. H. Trinh T. H. Meesad P. Nguyen T. T. (2022). Improved YOLO object detection algorithm to detect ripe pineapple phase. J. Intelligent Fuzzy Syst. 43, 1365–1381. doi: 10.3233/JIFS-213251

[B17] Deng F. He Z. Fu L. Chen J. Li N. Chen W. . (2025). A new maturity recognition algorithm for Xinhui citrus based on improved YOLOv8. Front. Plant Sci. 16, 1472230. doi: 10.3389/fpls.2025.1472230 39980487 PMC11841428

[B16] Deng B. Lu Y. Li Z. (2024). Detection, counting, and maturity assessment of blueberries in canopy images using YOLOv8 and YOLOv9. Smart Agric. Technol. 9, 100620. doi: 10.1016/j.atech.2024.10062042574925

[B18] Diep T. T. Pook C. Yoo M. J. Y. (2020). Physicochemical properties and proximate composition of tamarillo (*Solanum betaceum* Cav.) fruits from New Zealand. J. Food Compos. Anal. 92, 103563. doi: 10.1016/j.jfca.2020.10356342574925

[B19] Fan D.-P. Ji G.-P. Cheng M.-M. Shao L. (2022). Concealed object detection. IEEE Trans. Pattern Anal. Mach. Intell. 44, 6024–6042. doi: 10.1109/TPAMI.2021.3085766 34061739

[B20] Faria S. E. S. Azevedo A. M. Rabelo N. G. Anastácio V. Z. Maciel V. D. M. Matos D. V. . (2025). Advanced phenotyping in tomato fruit classification through artificial intelligence. Sci. Agric. 82, e20240115. doi: 10.1590/1678-992x-2024-011541099703

[B21] Gao X. Ding J. Bie M. Yu H. Shen Y. Zhang R. . (2025). YOLOv8n-FDE: An efficient and lightweight model for tomato maturity detection. Agronomy 15, 1899. doi: 10.3390/agronomy1508189930654563

[B22] Garillos-Manliguez C. A. Chiang J. Y. (2021). Multimodal deep learning and visible-light and hyperspectral imaging for fruit maturity estimation. Sensors 21, 1288. doi: 10.3390/s21041288 33670232 PMC7916978

[B23] Gelman A. Hill J. (2006). Data Analysis Using Regression and Multilevel/Hierarchical Models (New York: Cambridge University Press). doi: 10.1017/CBO9780511790942

[B24] Ghiasi G. Cui Y. Srinivas A. Qian R. Lin T.-Y. Cubuk E. D. . (2020). Simple copy-paste is a strong data augmentation method for instance segmentation. arXiv. doi: 10.48550/ARXIV.2012.07177

[B25] Gong X. Wu Q. (2025). Fruit detection methods based on deep learning in agricultural planting: A systematic literature review. IEEE Access 13, 96092–96110. doi: 10.1109/ACCESS.2025.357336425079929

[B26] Hao Y. Rao L. Fu X. Zhou H. Li H. (2025). Tomato ripening detection in complex environments based on improved BiAttFPN fusion and YOLOv11-SLBA modeling. Agriculture 15, 1310. doi: 10.3390/agriculture1512131030654563

[B27] Hastie T. Tibshirani R. Friedman J. (2009). The Elements of Statistical Learning Data Mining, Inference, and Prediction (New York, NY: Springer).

[B28] Hu C. Gao X. Dou K. Zhu C. Zhou Y. Hu Z. (2023). Physiological and metabolic changes in tamarillo (*Solanum betaceum*) during fruit ripening. Molecules 28, 1800. doi: 10.3390/molecules28041800 36838788 PMC9966127

[B30] Huang Y.-H. Huang C.-Y. (2024). Anti-skin aging and cytotoxic effects of methanol-extracted *Solanum betaceum* red fruit seed extract on Ca9-22 gingival carcinoma cells. Plants 13, 2215. doi: 10.3390/plants13162215 39204651 PMC11360763

[B29] Huang W. Wang J. Zhang S. Chen T. Zhao C. Huang G. . (2026). MEGNet: A multi-scale edge geometry-aware network for green plum detection in picking orchard environment. Horticulturae 12, 682. doi: 10.3390/horticulturae1206068230654563

[B31] HumanSignal (2025). Label-Studio 1.23.0 [Label-Studio 1.23.0]. Available online at: https://pypi.org/project/label-studio/ (Accessed June 26, 2026).

[B32] Imania H. A. N. Rachma A. F. Kurniawati L. A. Hidayati H. B. Wungu C. D. K. Nazmuddin M. (2024). Potential of *Solanum betaceum* to improve cognition: A systematic review of animal studies. J. Pharm. Pharmacognosy Res. 12, 1–13. doi: 10.56499/jppres23.1729_12.1.142602845

[B33] Isiaka F. (2024). An intelligent analytics for people detection using deep learning. 1–14. doi: 10.32388/5SRR92

[B34] Jia W. Liu J. Lu Y. Liu Q. Zhang T. Dong X. (2022). Polar-Net: Green fruit instance segmentation in complex orchard environment. Front. Plant Sci. 13, 1054007. doi: 10.3389/fpls.2022.1054007 36589132 PMC9796563

[B35] Jia W. Xu Y. Lu Y. Yin X. Pan N. Jiang R. . (2023). An accurate green fruits detection method based on optimized YOLOX-m. Front. Plant Sci. 14, 1187734. doi: 10.3389/fpls.2023.1187734 37223802 PMC10200941

[B36] Jocher G. Chaurasia A. Qiu J. (2023). YOLO by ultralytics. Available online at: https://github.com/ultralytics/ultralytics (Accessed July 2, 2026).

[B37] Kamat P. Gite S. Chandekar H. Dlima L. Pradhan B. (2025). Multi-class fruit ripeness detection using YOLO and SSD object detection models. Discover Appl. Sci. 7, 931. doi: 10.1007/s42452-025-07617-730311153

[B38] Kanna S. K. Ramalingam K. P P. R J. P.C. P. (2024). YOLO deep learning algorithm for object detection in agriculture: A review. J. Agric. Eng. 55. doi: 10.4081/jae.2024.1641

[B39] Karegowda A. G. D U H. Sheela S. J. (2024). “ Fruit classification and ripeness estimation using deep learning models”, in: 2024 8th International Conference on Computational System and Information Technology for Sustainable Solutions (CSITSS) ( IEEE), 1–6. doi: 10.1109/CSITSS64042.2024.10817041

[B40] Kim K. G. (2016). Book review: Deep learning. Healthcare Inf. Res. 22, 351. doi: 10.4258/hir.2016.22.4.351

[B41] Kohavi R. (1995). A study of cross-validation and bootstrap for accuracy estimation and model selection. 2, 1137–1145.

[B42] Lawal O. M. Zhao H. Zhu S. Chuanli L. Cheng K. (2024). Lightweight fruit detection algorithms for low‐power computing devices. IET Image Proc. 18, 2318–2328. doi: 10.1049/ipr2.13098

[B44] Lin Y. Huang Z. Liang Y. Liu Y. Jiang W. (2024). AG-YOLO: A rapid citrus fruit detection algorithm with global context fusion. Agriculture 14, 114. doi: 10.3390/agriculture1401011430654563

[B43] Lin X. Liao D. Du Z. Wen B. Wu Z. Tu X. (2025). SDA-YOLO: An object detection method for peach fruits in complex orchard environments. Sensors 25, 4457. doi: 10.3390/s25144457 40732584 PMC12298005

[B45] Liu Y. Yu Q. Geng S. Guo S. Liu L. (2025). SSViT-YOLOv11: Fusing lightweight YOLO & ViT for coffee fruit maturity detection. Front. Plant Sci. 16, 1691643. doi: 10.3389/fpls.2025.1691643 41404146 PMC12702924

[B46] Lu J. Yang R. Yu C. Lin J. Chen W. Wu H. . (2022). Citrus green fruit detection via improved feature network extraction. Front. Plant Sci. 13, 946154. doi: 10.3389/fpls.2022.946154 36578336 PMC9791251

[B47] Ma J. Li M. Fan W. Liu J. (2024). State-of-the-art techniques for fruit maturity detection. Agronomy 14, 2783. doi: 10.3390/agronomy1412278330654563

[B48] Ma N. Sun Y. Li C. Liu Z. Song H. (2025). AHG-YOLO: Multi-category detection for occluded pear fruits in complex orchard scenes. Front. Plant Sci. 16, 1580325. doi: 10.3389/fpls.2025.1580325 40487208 PMC12141327

[B49] MaChado A. M. R. Teodoro A. J. Mariutti L. R. B. Fonseca J. C. N. D. (2024). Tamarillo (*Solanum betaceum* Cav.) wastes and by-products: Bioactive composition and health benefits. Heliyon 10, e37600. doi: 10.1016/j.heliyon.2024.e37600 39309964 PMC11416485

[B50] Mi Z. Yan W. Q. (2024). Strawberry ripeness detection using deep learning models. Big Data Cogn. Computing 8, 92. doi: 10.3390/bdcc808009230654563

[B51] Mienye I. D. Swart T. G. (2024). A comprehensive review of deep learning: Architectures, recent advances, and applications. Information 15, 755. doi: 10.3390/info1512075530654563

[B52] Mishra R. K. Reddy G. Y. S. Pathak H. (2021). The understanding of deep learning: A comprehensive review. Math. Probl. Eng. 2021, 1–15. doi: 10.1155/2021/5548884

[B53] Murillo-Gómez P. A. Hoyos S R. Chavarriaga P. (2017). Organogénesis in-vitro using three tissues types of tree tomato [ *Solanum betaceum* (Cav.). Agronomía Colombiana 35, 5–11. doi: 10.15446/agron.colomb.v35n1.61330

[B54] Nath N. D. Behzadan A. H. Paal S. G. (2020). Deep learning for site safety: Real-time detection of personal protective equipment. Autom. Constr. 112, 103085. doi: 10.1016/j.autcon.2020.10308542574925

[B55] Ndisanze M. A. Koca I. (2023). Modelling, phytochemical, aroma and antioxidant capacity of tamarillo fruits dried with convective oven equipped with programmable logical control (PLC) at different temperatures: A comparative study. J. Food Meas. Charact. 17, 1438–1454. doi: 10.1007/s11694-022-01713-730311153

[B56] Patra G. K. Rao K. Marndi A. Chowdhury S. Rahaman J. Nandi S. . (2023). “ Deep learning methods for scientific and industrial research,” in Handbook of Statistics, vol. 48. ( Elsevier), 107–168. doi: 10.1016/bs.host.2022.12.002

[B57] Pérez-Pérez B. D. García Vázquez J. P. Salomón-Torres R. (2021). Evaluation of convolutional neural networks’ hyperparameters with transfer learning to determine sorting of ripe Medjool dates. Agriculture 11, 115. doi: 10.3390/agriculture1102011530654563

[B58] Pugazhendi P. Badgujar C. M. Sapkota R. Dhillon R. S R. J J. J. S. . (2026). Advances in agricultural fruit detection using you only look once (YOLO) algorithm: A review. Smart Agric. Technol. 13, 101896. doi: 10.1016/j.atech.2026.10189642574925

[B59] Rajeev P. A. Dharewa V. Lakshmi D. Vishnuvarthanan G. Giri J. Sathish T. . (2025). Advancing e-waste classification with customizable YOLO based deep learning models. Sci. Rep. 15, 18151. doi: 10.1038/s41598-025-94772-x 40415121 PMC12104417

[B60] Rito M. Marques J. Da Costa R. M. F. Correia S. Lopes T. Martin D. . (2023). Antioxidant potential of tamarillo fruits—Chemical and infrared spectroscopy analysis. Antioxidants 12, 536. doi: 10.3390/antiox12020536 36830094 PMC9952541

[B61] Rohilla S. Mahanta C. L. (2024). Phytochemical composition and in-vitro bioaccessibility of phenolics in different varieties of tamarillo (*Solanum betaceum*) fruits: Effect of the high-pressure homogenization and ultrasonication. Food Chem. Adv. 4, 100673. doi: 10.1016/j.focha.2024.10067342574925

[B62] Sa I. Ge Z. Dayoub F. Upcroft B. Perez T. McCool C. (2016). DeepFruits: A fruit detection system using deep neural networks. Sensors 16, 1222. doi: 10.3390/s16081222 27527168 PMC5017387

[B63] Sapkota R. Flores-Calero M. Qureshi R. Badgujar C. Nepal U. Poulose A. . (2025). YOLO advances to its genesis: A decadal and comprehensive review of the You Only Look Once (YOLO) series. Artif. Intell. Rev. 58, 274. doi: 10.1007/s10462-025-11253-330311153

[B64] Sarker I. H. (2021). Deep learning: A comprehensive overview on techniques, taxonomy, applications and research directions. SN Comput. Sci. 2, 420. doi: 10.1007/s42979-021-00815-1 34426802 PMC8372231

[B65] Shorten C. Khoshgoftaar T. M. (2019). A survey on image data augmentation for deep learning. J. Big Data 6, 60. doi: 10.1186/s40537-019-0197-0 34306963 PMC8287113

[B66] Soto-Osorio D. Sidorov G. Chanona-Hernández L. López-Ramírez B. C. (2024). Identification of scientific texts generated by large language models using machine learning. Computers 13, 346. doi: 10.3390/computers1312034630654563

[B68] Talaei Khoei T. Ould Slimane H. Kaabouch N. (2023). Deep learning: Systematic review, models, challenges, and research directions. Neural Comput. Appl. 35, 23103–23124. doi: 10.1007/s00521-023-08957-430311153

[B70] Tang Y. Qiu J. Zhang Y. Wu D. Cao Y. Zhao K. . (2023). Optimization strategies of fruit detection to overcome the challenge of unstructured background in field orchard environment: A review. Precis. Agric. 24, 1183–1219. doi: 10.1007/s11119-023-10009-930311153

[B69] Tang J. Yu Z. Shao C. (2025). Hybrid attention transformer integrated YOLOV8 for fruit ripeness detection. Sci. Rep. 15, 22652. doi: 10.1038/s41598-025-04184-0 40592947 PMC12219097

[B71] Tian Y. Yang G. Wang Z. Wang H. Li E. Liang Z. (2019). Apple detection during different growth stages in orchards using the improved YOLO-V3 model. Comput. Electron. Agric. 157, 417–426. doi: 10.1016/j.compag.2019.01.01242574925

[B73] Wang C.-Y. Bochkovskiy A. Liao H.-Y. M. (2021). “ Scaled-YOLOv4: Scaling cross stage partial network”, in: 2021 IEEE/CVF Conference on Computer Vision and Pattern Recognition (CVPR) ( IEEE), 13024–13033. doi: 10.1109/CVPR46437.2021.01283

[B74] Wang C.-Y. Bochkovskiy A. Liao H.-Y. M. (2023). “ YOLOv7: Trainable bag-of-freebies sets new state-of-the-art for real-time object detectors”, in: 2023 IEEE/CVF Conference on Computer Vision and Pattern Recognition (CVPR) ( IEEE), 7464–7475. doi: 10.1109/CVPR52729.2023.00721

[B75] Wang G. Gao Y. Xu F. Sang W. Han Y. Liu Q. (2025). A banana ripeness detection model based on improved YOLOv9c multifactor complex scenarios. Symmetry 17, 231. doi: 10.3390/sym1702023130654563

[B76] Wang J. Guo Y. Tan X. Lan Y. Han Y. (2025). Enhancing green guava segmentation with texture consistency loss and reverse attention mechanism under complex background. Comput. Electron. Agric. 235, 110308. doi: 10.1016/j.compag.2025.11030842574925

[B72] Wang C. Han Q. Li C. Zou T. Zou X. (2024). Fusion of fruit image processing and deep learning: A study on identification of citrus ripeness based on R-LBP algorithm and YOLO-CIT model. Front. Plant Sci. 15, 1397816. doi: 10.3389/fpls.2024.1397816 38903428 PMC11188418

[B78] Wang X. Huang Y. Wei S. Xu W. Zhu X. Mu J. . (2025). ELD-YOLO: A lightweight framework for detecting occluded mandarin fruits in plant research. Plants 14, 1729. doi: 10.3390/plants14111729 40508403 PMC12158239

[B77] Wang J. Shang Y. Zheng X. Zhou P. Li S. Wang H. (2024). GreenFruitDetector: Lightweight green fruit detector in orchard environment. PloS One 19, e0312164. doi: 10.1371/journal.pone.0312164 39541312 PMC11563454

[B79] Wei J. Ni L. Luo L. Chen M. You M. Sun Y. . (2024). GFS-YOLO11: A maturity detection model for multi-variety tomato. Agronomy 14, 2644. doi: 10.3390/agronomy1411264430654563

[B80] Xiao B. Nguyen M. Yan W. Q. (2023). Fruit ripeness identification using YOLOv8 model. Multimedia Tools Appl. 83, 28039–28056. doi: 10.1007/s11042-023-16570-930311153

[B81] Xu D. Ren R. Zhao H. Zhang S. (2024). Intelligent detection of muskmelon ripeness in greenhouse environment based on YOLO-RFEW. Agronomy 14, 1091. doi: 10.3390/agronomy1406109130654563

[B82] Yang X. Zhao W. Wang Y. Yan W. Q. Li Y. (2024). Lightweight and efficient deep learning models for fruit detection in orchards. Sci. Rep. 14, 26086. doi: 10.1038/s41598-024-76662-w 39477987 PMC11525789

[B500] Ye R. Gao Q. Qian Y. Sun J. Li T. (2024). Improved YOLOv8 and SAHI model for the collaborative detection of small targets at the micro scale: a case study of pest detection in tea. Agronomy 14 (5), 1034. doi: 10.3390/agronomy14051034

[B83] Yu H. Qian C. Chen Z. Chen J. Zhao Y. (2025). Ripe-detection: A lightweight method for strawberry ripeness detection. Agronomy 15, 1645. doi: 10.3390/agronomy1507164530654563

[B84] Yu Y. Zhang K. Yang L. Zhang D. (2019). Fruit detection for strawberry harvesting robot in non-structural environment based on Mask-RCNN. Comput. Electron. Agric. 163, 104846. doi: 10.1016/j.compag.2019.06.00142574925

[B87] Zhang W. (2023). A fruit ripeness detection method using adapted deep learning-based approach. Int. J. Advanced Comput. Sci. Appl. 14, 1163–1169. doi: 10.14569/IJACSA.2023.01409121

[B85] Zhang H. Cisse M. Dauphin Y. N. Lopez-Paz D. (2017). *mixup: Beyond empirical risk minimization* (Versión 2). Arxiv. 1–13. doi: 10.48550/ARXIV.1710.09412

[B86] Zhang R. Dong W. Hou P. Li H. Han X. Chen Q. . (2025). YOLOv11-BSD: Blueberry maturity detection under simulated nighttime conditions evaluated with causal analysis. Smart Agric. Technol. 12, 101314. doi: 10.1016/j.atech.2025.10131442574925

[B88] Zhou X. Hu X. Sun J. (2025). A review of fruit ripeness recognition methods based on deep learning. Cyber-Phys. Syst. 11, 508–542. doi: 10.1080/23335777.2025.246763937339054

[B89] Zhu F. Wang S. Liu M. Wang W. Feng W. (2025). A lightweight algorithm for detection and grading of olive ripeness based on improved YOLOv11n. Agronomy 15, 1030. doi: 10.3390/agronomy1505103030654563

